# Supplementary material for: Exploring exon excision as a therapeutic intervention strategy for the future treatment of ADGRV1-associated retinitis pigmentosa
Source: Mol Ther Nucleic Acids. 2025 Sep 3;36(4):102702. doi: 10.1016/j.omtn.2025.102702 (PMC12481715; doi:10.1016/j.omtn.2025.102702)
Supplement: Document S1. Figure S1 and Tables S1–S3 [file mmc1.pdf]

OMTN, Volume *36*

Supplemental information

Exploring exon excision as a therapeutic intervention strategy for the future treatment of *ADGRV1*-associated retinitis pigmentosa

Merel Stemerdink, Lucija Malinar, Sanne Broekman, Theo Peters, Iris Ensink, Maryna V. Ivanchenko, Hanka Venselaar, Hannie Kremer, Erik de Vrieze, and Erwin van Wijk

SUPPLEMENTAL MATERIAL

Table S1: sgRNA target sequences

**sgRNA target:**		**Sequence (5' → 3')**
Zebrafish *adgrv1* (ENSDART00000008043.9)	intron 8	GGACTTTGCTTGTACCACAG
Zebrafish *adgrv1* (ENSDART00000008043.9)	intron 9	GGTAAACTGTTCAAACCATA
Zebrafish *adgrv1* (ENSDART00000008043.9)	intron 39	AGCGAGGGAAGTGGAAATTG
Zebrafish *adgrv1* (ENSDART00000008043.9)	intron 42	GTCAGAGGTGATTCAGAAAA
Human *ADGRV1* (ENST00000405460.9)	intron 39	ATGCTTATCACGTCTGAATA
Human *ADGRV1* (ENST00000405460.9)	intron 42	CAGTATACCTCAACACTATG

Table S2: Primers used for genomic PCR and RT-PCR analysis of zebrafish samples

	**Primer name**	**Primer sequence (5' → 3')**
***adgrv1**$^{\Delta exon9}$* **genotyping**	*adgrv1* wild type forward	TATGGTCGATTTGGCTTTCATCC
	adgrv1 wild type reverse*	TGTGTTGTCCCAACTGTAATGTC
	adgrv1$^{\Delta exon9}$ forward	GTTTTGTGGTCCAGGGTCAC
	adgrv1$^{\Delta exon9}$ reverse*	TGTGTTGTCCCAACTGTAATGTC
***adgrv1**$^{\Delta exon40\text{-}42}$* **genotyping**	*adgrv1*$^{\Delta exon40\text{-}42}$ forward	CAGAAAGCAGGCTATGTC
	adgrv1$^{\Delta exon40\text{-}42}$ reverse	CATGCCCATCGTCATTAG
***adgrv1**$^{\Delta exon9}$* **transcript analysis**	*adgrv1* exon 7-11 forward	GTGCAGATCAAGATCTCCCGT
	adgrv1 exon 7-11 reverse	GTTGTCCACATTTGTCCTGTCT
***adgrv1**$^{\Delta exon40\text{-}42}$* **transcript analysis**	*adgrv1* exon 35-45 forward	GACGGCTCCTGCTATTGGAA
	adgrv1 exon 35-45 reverse	CCTCCACTGTCATGGTGTCC

*Primers are identical

Table S3: Primers used for generation PX459 plasmid and genomic PCR on HEK293T samples

	Primer name	Primer sequence (5' → 3')
**sgRNA integration in PX459**	PX459 *ADGRV1* intron 39 forward	CACCGATGCTTATCACGTCTGAATA
	PX459 *ADGRV1* intron 39 reverse	AAACTATTCAGACGTGATAAGCATC
	PX459 *ADGRV1* intron 42 forward	CACCGCAGTATACCTCAACACTATG
	PX459 *ADGRV1* intron 42 reverse	AAACCATAGTGTTGAGGTATACTGC
**Editing efficiency amplicon**	*ADGRV1* intron 39 forward-1	TACTCCTTCCAACTGTCCCC
	ADGRV1 intron 39 reverse-1	TTCAATTACACCTCGGGCCC
	ADGRV1 intron 42 forward-1	GCCTCTCACGTGTGTATGTTG
	ADGRV1 intron 42 reverse-1	GGATTGAATGCTGACTGACCC
**Exon excision amplicon**	*ADGRV1* intron 39 forward-2	CAAACTCCTCTTTCAGTGTG
	ADGRV1 intron 42 reverse-2	TTCATACAACGCTTTACCAC
***GAPDH* loading control**	*GAPDH* exon 4 forward	CATCTTCCAGGAGTGAGTGG
	GAPDH intron 4 reverse	CCATATTGAGGGACACAAGG

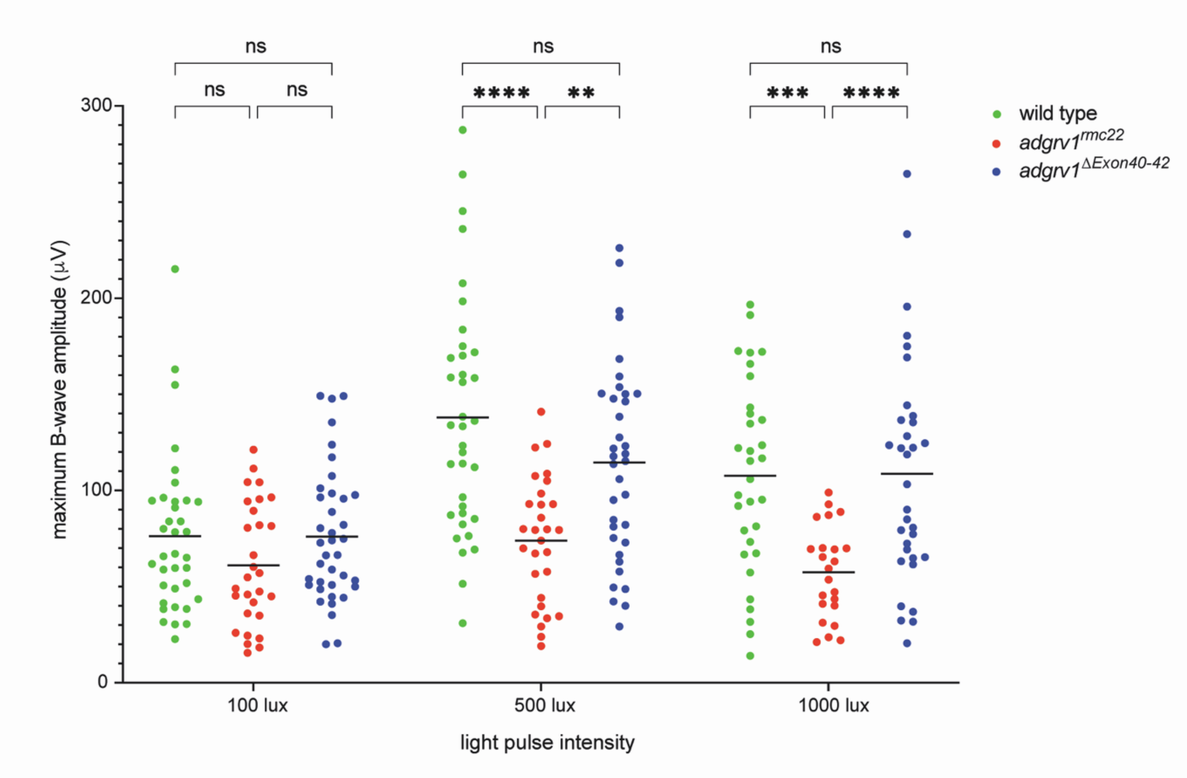

Figure S1: Electroretinogram maximum B-wave amplitudes plotted per light intensity. Maximum B-wave amplitudes following a light stimulus of 100, 500 and 1000 lux. The mean maximum B-wave amplitude of *adgrv1*$^{\Delta exon40\text{-}42}$ larvae is similar to those observed in wild types, whereas *adgrv1*^{rmc22} larvae show a decreased maximum B-wave amplitude (**, p = 0.0012; ***, p = 0.0001; ****, p < 0.0001; one-way ANOVA followed by Tukey's multiple comparison test).
