# Supplementary material for: Exploring exon excision as a therapeutic intervention strategy for the future treatment of ADGRV1-associated retinitis pigmentosa
Source: Mol Ther Nucleic Acids. 2025 Sep 3;36(4):102702. doi: 10.1016/j.omtn.2025.102702 (PMC12481715; doi:10.1016/j.omtn.2025.102702)
Supplement: Document S2. Article plus Supplemental information [file mmc2.pdf]

Molecular Therapy
Nucleic Acids
Original Article

Exploring exon excision as a therapeutic intervention strategy for the future treatment of *ADGRV1*-associated retinitis pigmentosa

Merel Stemerdink,[1,2] Lucija Malinar,[3] Sanne Broekman,[1] Theo Peters,[1] Iris Ensink,[4] Maryna V. Ivanchenko,[5] Hanka Venselaar,[3] Hannie Kremer,[1,4] Erik de Vrieze,[1] and Erwin van Wijk[1]

[1]Department of Otorhinolaryngology, Radboud University Medical Center, 6525 GA Nijmegen, the Netherlands; [2]Donders Institute for Brain, Cognition and Behaviour, Radboud University Medical Center, 6525 GA Nijmegen, the Netherlands; [3]Center for Molecular and Biomolecular Informatics, Radboud University Medical Center, 6525 GA Nijmegen, the Netherlands; [4]Department of Human Genetics, Radboud University Medical Center, 6525 GA Nijmegen, the Netherlands; [5]Department of Neurobiology, Harvard Medical School, Boston, MA, USA

Given the lack of treatment options for *ADGRV1*-associated retinitis pigmentosa (RP), we evaluated exon excision as a potential therapeutic strategy. To enable careful selection of target exons, *in silico* analyses of the ADGRV1 protein domain structure were performed using AlphaFold2. These revealed a more complex ADGRV1 domain architecture compared to previous 2D assessments, indicating the presence of a larger number of Calxβ domains as well as a previously unidentified cysteine-rich domain. Based on 2D and 3D protein modeling and the presence of loss-of-function mutations, we selected two targets for exon excision: exon 9 and exons 40–42. To assess functional consequences, we generated zebrafish mutants lacking these exons using CRISPR-Cas9. While exon 9 excision failed to result in Adgrv1 expression, excision of exons 40–42 preserved protein expression, enabling proper USH2 protein complex assembly, rhodopsin trafficking, and electroretinogram (ERG) responses comparable to wild type. Additionally, CRISPR-Cas9-mediated removal of *ADGRV1* exons 40–42 in HEK293T cells demonstrated the feasibility of this approach in the context of the human genome. These findings suggest that a carefully designed exon excision strategy may offer a viable treatment for *ADGRV1*-associated RP. Furthermore, they highlight the importance of 3D protein modeling in predicting structural consequences of exon excision and optimizing therapeutic design.

INTRODUCTION

Usher syndrome is an autosomal recessively inherited disorder characterized by the combination of sensorineural hearing loss and progressive loss of vision due to retinitis pigmentosa (RP). The disorder exhibits clinical and genetic diversity, with different (sub)types classified based on the clinical presentation and the gene that is involved.[1,2] The *ADGRV1* gene is one of the eleven genes associated with Usher syndrome, and pathogenic variants in this gene have been identified as the underlying cause of Usher syndrome type 2C (USH2C).[3] It is estimated that approximately 40,000 individuals worldwide are affected by USH2C. These individuals typically experience congenital hearing impairment, while the initial symptoms of RP manifest during the second decade of life. The initial sign of RP includes night blindness, followed by progressive loss of peripheral vision that often leads to central vision impairment and functional blindness by the fifth or sixth decade of life.[4] While hearing aids or cochlear implants may alleviate hearing loss, there are currently no treatment options for *ADGRV1*-associated RP. However, an early genetic diagnosis after a failed neonatal hearing test offers a window of opportunity to initiate therapeutic interventions before the manifestation of visual dysfunction. Therefore, this study aimed to develop a therapeutic strategy for the future treatment of *ADGRV1*-associated RP.

ADGRV1, previously referred to as *MASS1*, *VLGR1*, and *GPR98*, encodes the adhesion G-protein-coupled receptor v1 (ADGRV1). To date, three distinct transcript isoforms have been identified, resulting from alternative splicing events.[3,5] With a sequence length of 19.6 kb and a total of 90 exons, the largest transcript (isoform B) encodes a protein of 6,306 amino acids, making it the largest cell surface protein in man. This protein is characterized by an extensive extracellular region composed of numerous predicted Calxβ domains, which is followed by a seven-pass transmembrane region that anchors the protein in the cell membrane. In the retina, ADGRV1 is localized at the periciliary membrane and connecting cilium of photoreceptor cells, where it interacts with usherin (USH2A) and whirlin (USH2D) to form the USH2 protein complex.[6] This complex facilitates the transport of trans-Golgi-derived vesicles containing essential components for the proper development and maintenance of the photoreceptor outer segments. Cargo vesicles are docked at the photoreceptor periciliary membrane, after which proteins are handed over

Received 18 April 2025; accepted 2 September 2025;
https://doi.org/10.1016/j.omtn.2025.102702.

Correspondence: Erwin van Wijk, Department of Otorhinolaryngology, Radboud University Medical Center, 6525 GA Nijmegen, the Netherlands.
E-mail: erwin.vanwyk@radboudumc.nl

Published by Elsevier Inc. on behalf of The American Society of Gene and Cell Therapy.
This is an open access article under the CC BY license (http://creativecommons.org/licenses/by/4.0/).

to the intraflagellar transport system for further distribution to the outer segments.[6] Consequently, the disruption of this protein complex due to the loss of one of the USH2 proteins results in the development of RP.

The large coding sequence of *ADGRV1* presents challenges for gene augmentation strategies, as it surpasses the packaging capacity of currently used viral vehicles for gene delivery.[7,8] As an alternative, mutation- or exon-specific strategies such as the development of antisense oligonucleotide (ASO)-induced splice modulation therapies for inherited retinal dystrophies (IRDs) have emerged as a promising therapeutic option. ASO-based approaches are aimed at restoring protein expression and function by correcting aberrant pre-mRNA splicing or by removing native exons. For instance, ASOs can block the binding of splicing factors to cryptic splice sites activated by intronic mutations, thereby preventing inclusion of pseudoexons. Alternatively, they may induce skipping of native exons flanking frame-shifting genomic deletions to restore the open reading frame (ORF) or promote removal of in-frame exons that harbor (recurrent) loss-of-function mutations. Finally, ASOs can also be applied to promote the inclusion of native exons that are skipped during pre-mRNA splicing as a consequence of pathogenic variants. In previous work, we presented ultevursen, an ASO designed to induce skipping of *USH2A* exon 13 for the treatment of *USH2A*-associated RP caused by pathogenic variants in exon 13,[9] which is currently being evaluated in a phase 2b clinical trial (Trial #NCT06627179). To increase the chances of native exon skipping being therapeutically effective, it is preferable for the target exon (s) to encode complete protein domain(s), in order to avoid compromising protein folding and overall structural integrity. The exon skipping approach is especially compelling for genes whose encoded proteins exhibit a repetitive domain architecture, such as *DMD*, *NOTCH3*, and *USH2A*. However, due to the limited number of in-frame exons encoding complete protein domains within genes, multi-exon skipping has emerged as an alternative strategy to expand therapeutic targets. This approach targets multiple exons that, upon skipping, give rise to a protein that is able to fold properly, as demonstrated in studies addressing Duchenne muscular dystrophy (*DMD*), CADASIL (*NOTCH3)*, Miyoshi-myopathy (*DYSF*), and USH2A (*USH2A*).[10–13]

Building upon the success of single- and multi-exon skipping as a promising treatment paradigm for *USH2A*-associated RP, and given the repetitive protein domain architecture of *ADGRV1*, we hypothesized that the exon skipping strategy could be extended to *ADGRV1*-associated RP. Analysis of the *ADGRV1* LOVD mutation database (https://databases.lovd.nl/shared/genes/GPR98) revealed the presence of multiple pathogenic variants in both exon 9 and exons 40–42. Based on allele frequencies in the general population for the reported pathogenic variants (gnomAD v.4.1.0), followed by calculations using the Hardy-Weinberg equilibrium, it is estimated that approximately 1,000 and 2,400 individuals worldwide, respectively, are affected by *ADGRV1*-associated RP caused by pathogenic variants in these exons. Based on these findings and careful 2D and 3D *in silico* protein domain analyses, we selected two targets for exon skipping. *ADGRV1* exon 9 was selected using SMART-based 2D protein domain predictions,[14] whereas *ADGRV1* exons 40–42 were identified as a promising target for exon skipping based on 3D structural modeling using a combination of resolved crystallized structures and AlphaFold2 predictions.[15] Next, to evaluate the functional consequences of exon skipping, we utilized the zebrafish model. We previously demonstrated the value of this model by generating $adgrv1^{rmc22}$ mutant zebrafish as a model for *ADGRV1*-associated retinal dysfunction.[16] Employing CRISPR-Cas9 genome editing, we now excised the orthologous exon 9 or exons 40–42 from the zebrafish genome, creating the $adgrv1^{\Delta exon9}$ and $adgrv1^{\Delta exon40\text{-}42}$ zebrafish lines. These lines were phenotypically compared to wild type and $adgrv1^{rmc22}$ zebrafish. While excision of exon 9 failed to result in Adgrv1 expression in zebrafish, the excision of exons 40–42 did not affect the expression and localization of Adgrv1 and its USH2 protein interaction partners in photoreceptor cells. Furthermore, in $adgrv1^{\Delta exon40\text{-}42}$ zebrafish, the rhodopsin trafficking and the electroretinogram (ERG) traces were indistinguishable from wild type. To further translate these findings into a potential therapeutic application, we employed CRISPR-Cas9 genome editing to successfully excise *ADGRV1* exons 40–42 in a human HEK293T cell line, demonstrating the feasibility of translating this approach into a future application in man. Collectively, our findings indicate that excision of *ADGRV1* exons 40–42 represents a promising therapeutic strategy for *ADGRV1*-associated RP and demonstrate how 3D structural modeling can facilitate the identification of additional target regions suitable for therapeutic exon excision or skipping approaches.

RESULTS

Analysis of human and zebrafish ADGRV1 protein domain architecture

Given that the zebrafish model will be used in this study to assess the therapeutic effects of exon excision, we first conducted a thorough comparison of the human and zebrafish ADGRV1 protein domain architecture prior to selecting targets for genomic exon excision. This was done to identify *ADGRV1* exons in both species that, when excised, would not compromise protein folding and therefore functionality. Furthermore, we also assessed potential similarities and differences in ADGRV1 protein folding between the two species, to better understand how findings from zebrafish studies may translate to a potential application in man. The full-length ADGRV1 (isoform B) protein comprises 6,306 amino acids in man and 6,199 amino acids in zebrafish. 2D protein domain analyses using SMART[14] revealed that both proteins exhibit a highly similar repetitive domain structure (Figure 1A). The 3D models of ADGRV1 that were generated using AlphaFold2[15] provided important structural insights that were not evident from the 2D domain analyses (Figure 1B). Specifically, the 3D models revealed a previously undetected cysteine-rich domain that is conserved between both species and showed that human and zebrafish ADGRV1 are built up by 39 Calxβ domains, instead of the previously predicted 35 Calxβ domains in the 2D representations. Additionally, the 3D modeling demonstrated that the

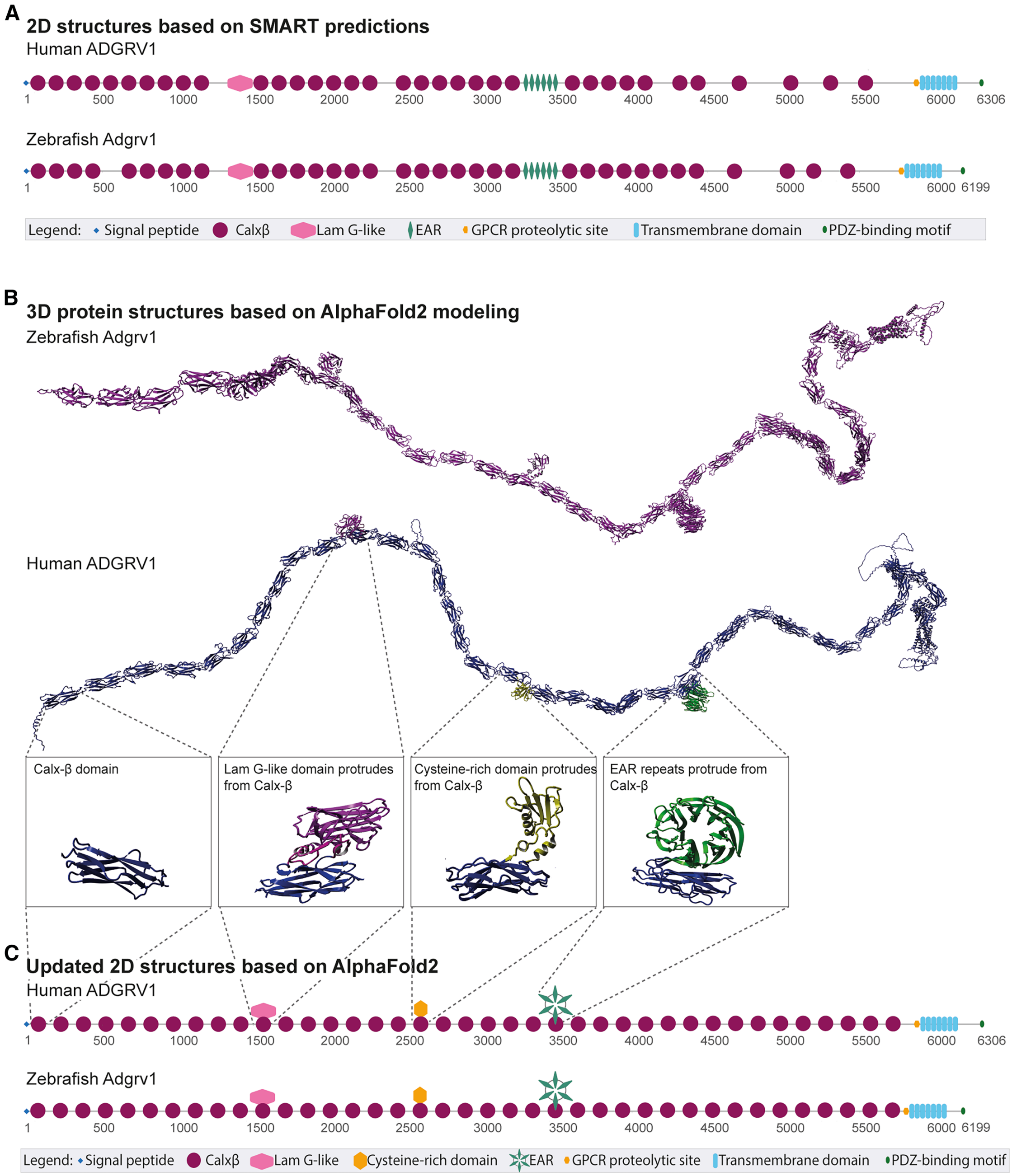

Figure 1. *In silico* 3D structural modeling of the ADGRV1 protein identifies a previously undetected cysteine-rich domain and additional Calxβ domains compared to previous 2D-based models

(A) Schematic representation of the protein domain structure of the large isoform (isoform B) of human and zebrafish ADGRV1, based on 2D-SMART predictions. Both proteins exhibit a repetitive domain architecture, comprising a signal peptide, 35 Ca^{2+}-binding calcium exchanger β domains (Calxβ), a laminin G-like domain (LamG-like), 6 epilepsy-associated repeats (EAR), a G-protein-coupled receptor (GPCR) proteolytic site, a seven-transmembrane region, and an intracellular region with a C-terminal class I PDZ-binding motif. Numbers indicate amino acid positions. (B) *In silico* 3D modeling of zebrafish (magenta) and human (blue) ADGRV1, based on AlphaFold2 predictions, suggests a more complex domain architecture than 2D-SMART-based models. 3D models revealed additional Calxβ domains as compared to the SMART-based predictions (39 instead of the previously predicted 35 Calxβ domains) and the presence of a previously unidentified cysteine-rich domain. Additionally, the 3D models reveal that the LamG-like domain, the cysteine-rich domain, and the EAR repeats protrude from the Calxβ domains, rather than existing as solitary domain structures that are separated by other (types of) domains. (C) Updated 2D schematic representation of human and zebrafish ADGRV1 (isoform B) based on novel 3D protein modeling.

laminin G-like domain, the cysteine-rich domain, and the epilepsy-associated repeats (EAR) protrude from Calxβ domains, rather than existing as solitary domain structures that are separated by other (types of) domains. Notably, these protruding domains interrupt the continuous amino acid sequence encoding the Calxβ domain from which they extend, splitting it into two segments that together reassemble into a functional Calxβ domain. Based on these structural insights, revised 2D schematic representations were generated to accurately depict the domain architectures of the human and zebrafish ADGRV1 proteins (Figure 1C).

ADGRV1 exon 9 and exons 40–42 selection as targets for exon excision therapy

Considering the discrepancies between the previously reported ADGRV1 domain architecture based on 2D prediction models,[14] and the updated models derived from our 3D structural protein modeling, we selected two potential targets for exon excision—one based on the SMART-predicted 2D structure and one based on 3D models.

First, based on the SMART-predicted 2D structure, *ADGRV1* exon 9 (330 nucleotides both in man and zebrafish) was selected as a target. Excision of this exon is expected to preserve the open reading frame and to result in the loss of a single Calxβ domain in the human ADGRV1 protein, with no predicted changes to the domain structure in zebrafish Adgrv1 (Figure 2A). Therefore, excision of exon 9 is anticipated to result in the production of a slightly shortened protein, without significantly disrupting the folding or functionality of ADGRV1. We also investigated the impact of *ADGRV1* exon 9 excision using the AlphaFold2 structural modeling approach (Figures 2B and 2C). In contrast to the SMART-based 2D predictions, the resulting 3D model revealed that exon 9 appears to encode two partial Calxβ domains in both man and zebrafish ADGRV1. Based on AlphaFold2 predictions, the removal of exon 9 results in a discontinuity in the Calxβ domain structure, leading to the production of a disordered protein structure. The functional consequences of this structural change remain uncertain and require further evaluation to determine the impact on ADGRV1 protein folding and functionality.

As a second target based on 3D structural modeling, we selected *ADGRV1* exons 40–42 (together 360 nucleotides) as a target for multi-exon excision (Figure 2D). Both SMART and AlphaFold2 modeling indicated that these exons encode two partial Calxβ domains in both man and zebrafish (Figures 2A and 2D). Although the loss of these partial domains initially suggested that this region might not be an ideal target for excision, AlphaFold2 predictions revealed that upon removal of exons 40–42, the remaining counterparts of Calxβ domains 21 and 22 assemble into a single-hybrid Calxβ domain (Figure 2E). This hybrid structure closely resembles native Calxβ domains in 3D conformation (Figure 2F), suggesting that the removal of exons 40–42 may be better tolerated than excision of exon 9. However, it remains to be evaluated whether this hybrid Calxβ domain can support normal ADGRV1 protein function.

Generation of zebrafish *adgrv1*$^{\Delta exon\ 9}$ and *adgrv1*$^{\Delta exons\ 40–42}$ deletion models using CRISPR-Cas9 technology

To evaluate the consequences of excising *ADGRV1* exon 9 and exons 40–42 on ADGRV1 protein function, we generated two stable zebrafish lines from which the genomic regions containing *adgrv1* exon 9 (330 nucleotides) or *adgrv1* exons 40–42 (360 nucleotides) were deleted. To generate these zebrafish lines, we injected a combination of two Cas9-sgRNA ribonucleoprotein complexes into single-cell-staged fertilized zebrafish embryos. One single guide RNA (sgRNA) was designed to target the intronic region upstream of the indicated exons, and the other sgRNA targeted the intronic region downstream of the indicated exons, to allow for the selective removal of these exons from the zebrafish genome (Figure 3A). The successful excision of the target exons was confirmed through genomic PCR and Sanger sequencing. Stable homozygous zebrafish lines harboring the excision of *adgrv1* exon 9 or exons 40–42, designated as *adgrv1*$^{\Delta exon9}$ and *adgrv1*$^{\Delta exon40\text{-}42}$ zebrafish, respectively, were subsequently bred from the germline-positive founder fish. Both the homozygous *adgrv1*$^{\Delta exon9}$ and *adgrv1*$^{\Delta exon40\text{-}42}$ zebrafish were viable and did not exhibit any apparent abnormalities in their development, morphology, or swimming behavior.

To assess the effect of exon 9 and exons 40–42 excisions on the *adgrv1* transcript levels, an RT-PCR analysis was conducted. cDNA, synthesized using total RNA extracted from homozygous *adgrv1*$^{\Delta exon9}$ and *adgrv1*$^{\Delta exon40\text{-}42}$ larvae, was used as a template for the amplification of the regions surrounding the excised target exons. This analysis revealed the presence of shortened PCR amplicons in both the *adgrv1*$^{\Delta exon9}$ and *adgrv1*$^{\Delta exon40\text{-}42}$ zebrafish samples, in the absence of any other detectable alternative splicing events (Figure 3B). Sanger sequencing indeed confirmed the absence of the anticipated target exons from *adgrv1* transcripts in *adgrv1*$^{\Delta exon9}$ and *adgrv1*$^{\Delta exon40\text{-}42}$ larvae.

Excision of *adgrv1* exons 40–42 in *adgrv1*$^{\Delta exon40\text{-}42}$ zebrafish does not affect Adgrv1 protein expression at the periciliary region of photoreceptor cells

Next, we determined whether excision of zebrafish *adgrv1* exon 9 and exons 40–42 resulted in the translation and correct localization of the shortened Adgrv1 proteins (Adgrv1$^{\Delta exon9}$ and Adgrv1$^{\Delta exon40\text{-}42}$) in the retina of homozygous *adgrv1*$^{\Delta exon9}$ and *adgrv1*$^{\Delta exon40\text{-}42}$ zebrafish. To this end, immunohistochemical analyses were performed, on retinal cryosections of wild-type, *adgrv1*^{rmc22}, *adgrv1*$^{\Delta exon9}$, and *adgrv1*$^{\Delta exon40\text{-}42}$ zebrafish larvae (5 days post-fertilization [dpf]) (Figure 4). In wild-type zebrafish, Adgrv1 is located at the periciliary region in close proximity to centrin. However, as previously observed, Adgrv1 is absent in photoreceptor cells of *adgrv1*^{rmc22} zebrafish, a null-mutant line that we previously generated as a model for *ADGRV1*-associated retinal dysfunction.[16] Remarkably, no Adgrv1 protein was detected in retinas of *adgrv1*$^{\Delta exon9}$ larvae. This suggests that the excision of exon 9 either disrupts the expression of Adgrv1 in the zebrafish retina or that it results in the formation of a highly unstable protein. In contrast, Adgrv1$^{\Delta exon40\text{-}42}$ was detected in retinas of *adgrv1*$^{\Delta exon40\text{-}42}$ larvae, with a subcellular localization comparable to

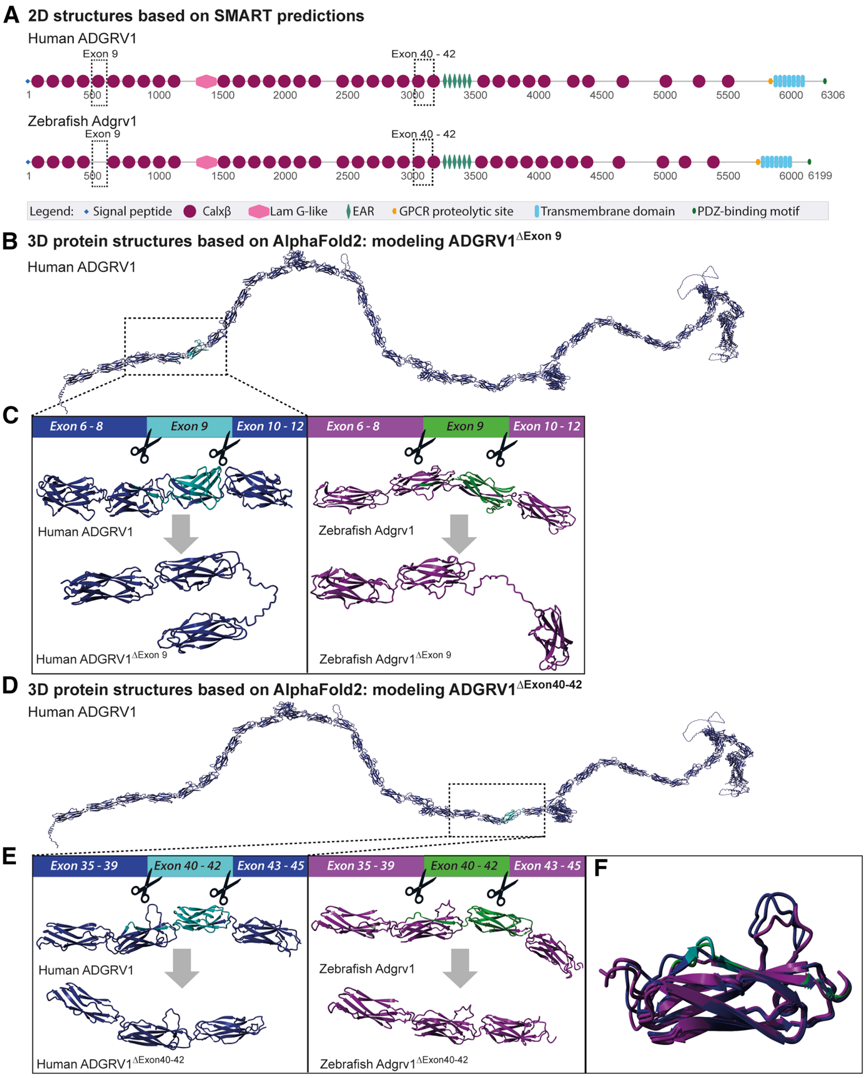

Figure 2. *In silico* modeling of the ADGRV1 protein domain architecture after the excision of *ADGRV1* exon 9 and exons 40–42

(A) Schematic representation of the protein domain structure of the large isoform (isoform B) of human and zebrafish ADGRV1, based on 2D-SMART protein predictions. Regions encoded by exon 9 and exons 40–42 are highlighted with dashed boxes. (B) *In silico* 3D modeling of human ADGRV1, based on AlphaFold2. The Calxβ domains encoded by *ADGRV1* exon 9 are depicted in cyan. (C) *In silico* 3D modeling of the protein domains encoded by exons 6–12 in man and zebrafish. The part encoded by exon 9 is depicted in cyan (human ADGRV1) or green (zebrafish Adgrv1). Removal of exon 9 is predicted to result in a discontinuity in the Calxβ domain structure. (D) *In silico* 3D modeling of human ADGRV1, based on AlphaFold2 predictions. The parts of Calxβ domains encoded by *ADGRV1* exons 40–42 are depicted in cyan. (E) *In silico* 3D modeling of the protein domains encoded by exons 35–45 in man and zebrafish. The part encoded by exons 40–42 is depicted in cyan (human ADGRV1) or green (zebrafish Adgrv1). Removal of exons 40–42 results in the production of a single-hybrid Calxβ domain, with a 3D structure highly similar to native Calxβ domains. (F) Structural comparison of native human and zebrafish Calxβ domains 21, along with the hybrid Calxβ domain resulting from the removal of exons 40–42 in both man and zebrafish. The superimposed image shows that the 3D structures of the hybrid human (blue) and zebrafish (magenta) Calxβ domains closely resemble the conformations of the native human Calxβ domain 21 (blue–cyan) and zebrafish Calxβ domain 21 (magenta–green).

wild types. This implies that the formation of a hybrid Calxβ domain, resulting from the excision of exons 40–42, does not interfere with Adgrv1 protein expression and localization.

The localization of the USH2 complex members, usherin and Whrnb, in the photoreceptor cells of *adgrv1*$^{\Delta exon40\text{-}42}$ zebrafish is comparable to that in wild-type zebrafish

Previous studies in man, mouse, and zebrafish have shown that ADGRV1 functions together with usherin and whirlin in a dynamic USH2 complex.[6,17] Our studies with the *adgrv1*^{rmc22} model have revealed that absence of Adgrv1 results in reduced levels of usherin and whirlin_b (Whrnb) at the periciliary region.[16] To assess whether Adgrv1$^{\Delta exon40\text{-}42}$ supports the proper assembly and localization of the USH2 protein complex, we performed immunohistochemical analyses on retinal cryosections of wild-type, *adgrv1*^{rmc22}, and *adgrv1*$^{\Delta exon40\text{-}42}$ zebrafish larvae, using antibodies against Whrnb and usherin (Figure 5). The intensity of the anti-Whrnb and anti-usherin fluorescence signals at the periciliary regions of larval photoreceptor cells was quantified. As previously observed, the expression levels of both Whrnb and usherin were significantly reduced in the *adgrv1*^{rmc22} mutants. However, the observed expression levels of both USH2 protein complex members in *adgrv1*$^{\Delta exon40\text{-}42}$ zebrafish larvae were comparable to wild types. This suggests that the Adgrv1$^{\Delta exon40\text{-}42}$ protein is indeed able to support the assembly of the USH2 protein complex at the photoreceptor periciliary region.

Excision of *adgrv1* exons 40–42 does not impair rhodopsin trafficking in zebrafish photoreceptor cells

The USH2 protein complex was previously shown to be involved in the intracellular transport of cargo vesicles, containing essential components for photoreceptor outer segment formation, maintenance, and function, such as photopigments (e.g., rhodopsin). Loss of either of the USH2 complex members has been shown to result in impaired rhodopsin trafficking (Figure 6A).[12,16,18] To evaluate if the Adgrv1$^{\Delta exon40\text{-}42}$ protein was able to facilitate normal rhodopsin transport, we examined the localization of rhodopsin in photoreceptor cells of 6 dpf wild-type, *adgrv1*^{rmc22}, and *adgrv1*$^{\Delta exon40\text{-}42}$

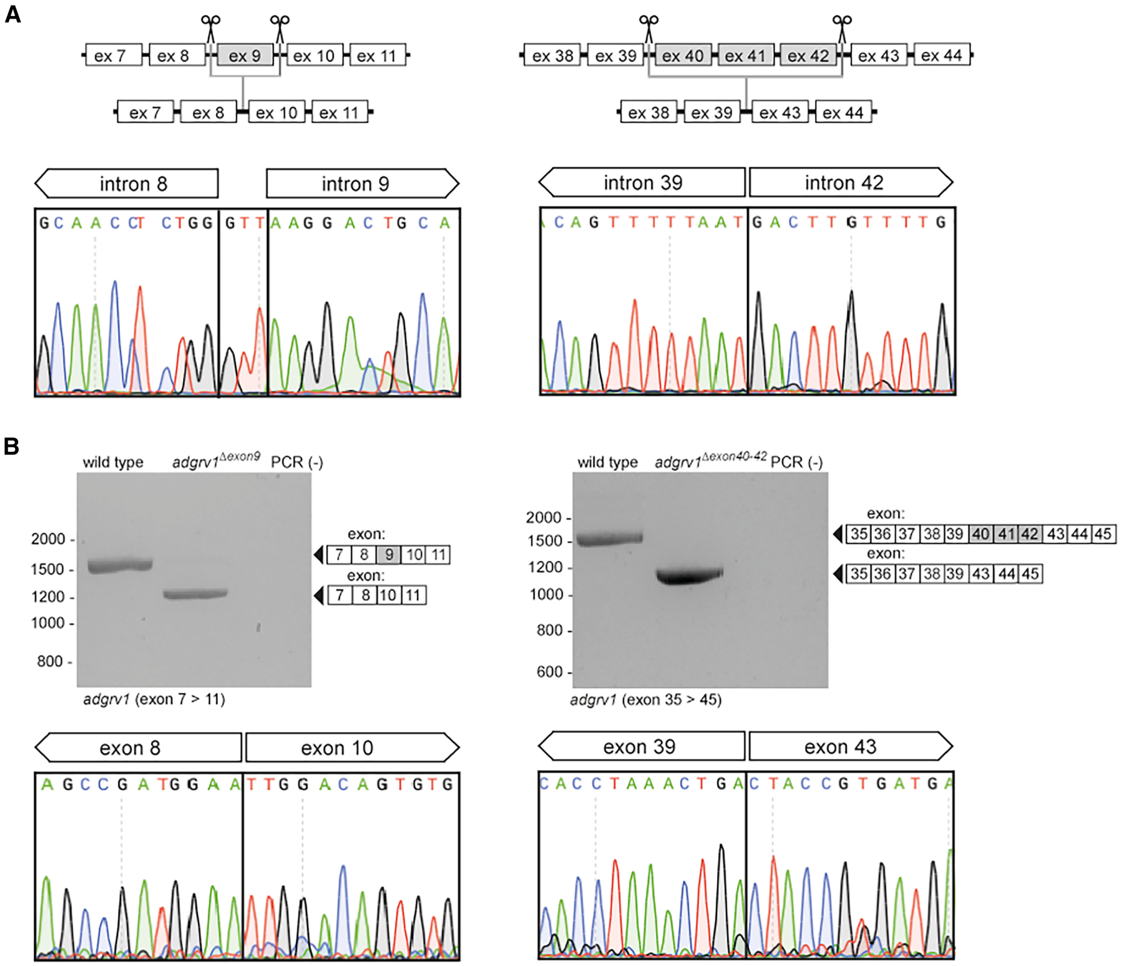

Figure 3. Design and characterization of the genetically modified *adgrv1*$^{\Delta exon9}$ and *adgrv1*$^{\Delta exon40\text{-}42}$ zebrafish lines

(A) Schematic representation illustrating the exon-excision approach. The anticipated excision of the target exons in injected embryos (1 day post-fertilization [dpf]) was confirmed by Sanger sequencing analysis. Excision of the genomic segment encompassing *adgrv1* exon 9 led to the incorporation of three additional nucleotides (GTT) at the repair site. (B) RT-PCR results demonstrate the absence of *adgrv1* exon 9 in *adgrv1*$^{\Delta exon9}$ larvae, as well as the absence of exons 40–42 in *adgrv1*$^{\Delta exon40\text{-}42}$ larvae (5 dpf). Sanger sequencing of RT-PCR amplicons confirmed the successful removal of the target exons from the *adgrv1* transcripts. PCR (−): negative PCR control.

zebrafish (Figure 6B). As observed in wild-type zebrafish, rhodopsin was also predominantly localized to the photoreceptor outer segments in *adgrv1*$^{\Delta exon40\text{-}42}$ zebrafish larvae, whereas a significantly higher number of photoreceptors with aberrant localization of rhodopsin was observed in *adgrv1*^{rmc22} larvae (Figure 6C) ($p < 0.0001$, one-way ANOVA followed by Tukey's multiple comparison test). This implies that Adgrv1$^{\Delta exon40\text{-}42}$ allows for normal ciliary trafficking of rhodopsin.

Excision of *adgrv1* exons 40–42 does not affect zebrafish visual function

To assess the effect of the excision of *adgrv1* exons 40–42 on visual function of zebrafish larvae, ERG recordings were performed on *adgrv1*$^{\Delta exon40\text{-}42}$ zebrafish larvae (7 dpf) following light stimuli of 100, 500, and 1,000 lux (n = 36 wild types, n = 36 *adgrv1*$^{\Delta exon40\text{-}42}$, and n = 29 *adgrv1*^{rmc22}, from three biological replicates). Across all light intensities, the maximum B-wave amplitudes of *adgrv1*$^{\Delta exon40\text{-}42}$ larvae were comparable to those recorded in age- and strain-matched wild-type controls (Figure S1). This is exemplified in Figure 7, which represents data from the 1,000 lux condition. In contrast, and consistent with previous findings,[16] *adgrv1*^{rmc22} larvae displayed significantly reduced maximum B-wave amplitudes, indicative of impaired retinal function ($p \leq 0.0001$, one-way ANOVA followed by Tukey's multiple comparison test).

Genomic CRISPR-Cas9-mediated excision of *ADGRV1* exons 40–42 from HEK293T cells

Building on the results obtained from the characterization of the *adgrv1*$^{\Delta exon40\text{-}42}$ zebrafish model—demonstrating proper localization of the Adgrv1$^{\Delta exon40\text{-}42}$ protein and its interaction partners, as well as

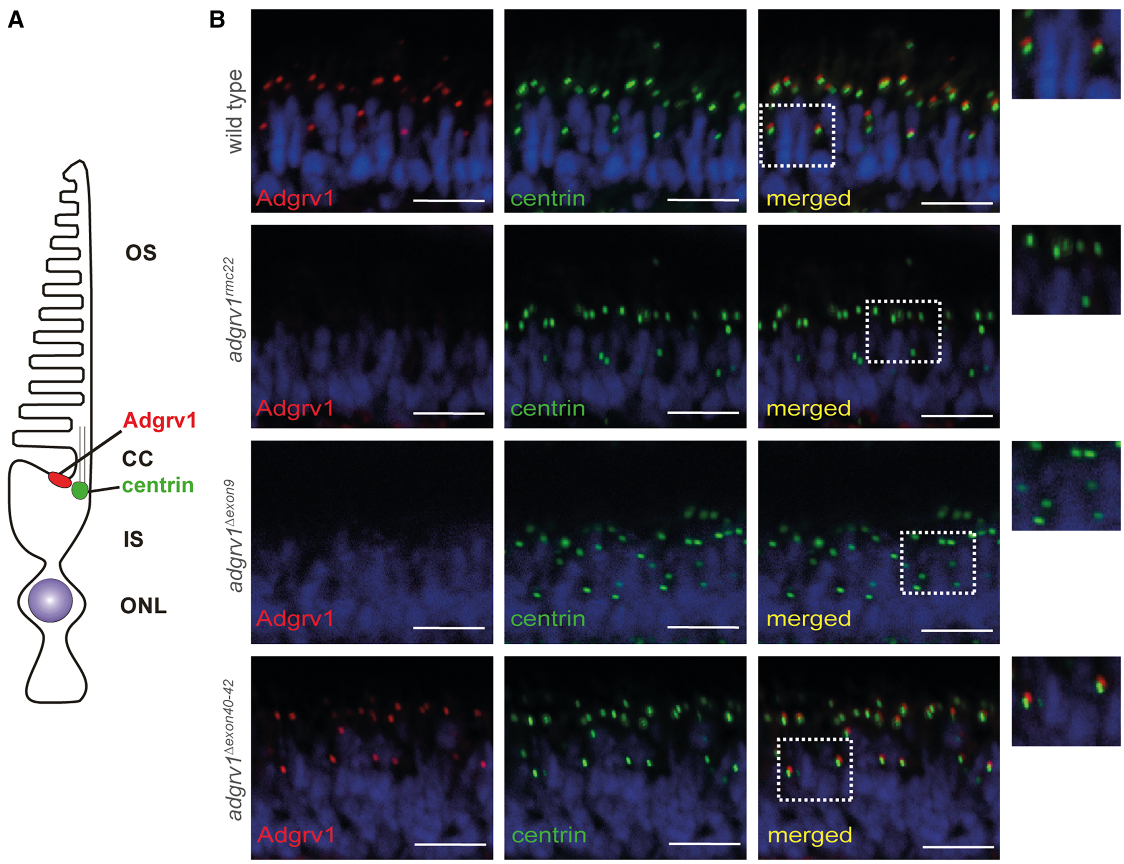

Figure 4. Analysis of Adgrv1 expression in retinal sections of wild-type, *adgrv1*^{rmc22}, *adgrv1*$^{\Delta exon9}$, and *adgrv1*$^{\Delta exon40\text{-}42}$ zebrafish

(A) Schematic representation of a zebrafish photoreceptor cell. OS, outer segment; CC, connecting cilium; IS, inner segment; ONL, outer nuclear layer. (B) Retinal cryosections of wild-type, homozygous *adgrv1*^{rmc22}, *adgrv1*$^{\Delta exon9}$, and *adgrv1*$^{\Delta exon40\text{-}42}$ zebrafish larvae (5 dpf) labeled with antibodies directed against Adgrv1 (red) and centrin (green). Nuclei are counterstained with DAPI (blue). No Adgrv1 signal was detected in *adgrv1*^{rmc22} and *adgrv1*$^{\Delta exon9}$ zebrafish retinae. In contrast, Adgrv1 was present and localized adjacent to centrin, a marker of the basal body and connecting cilium, in the retinas of wild-type and *adgrv1*$^{\Delta exon40\text{-}42}$ zebrafish larvae (n = 14 larvae per line, from two biological replicates for wild-type, *adgrv1*^{rmc22}, *adgrv1*$^{\Delta exon9}$, and *adgrv1*$^{\Delta exon40\text{-}42}$ larvae). Magnified images are shown at the right side. Scale bars, 10 μm.

normal rhodopsin trafficking and ERG responses comparable to wild types—we aimed to translate these findings into a CRISPR-Cas9-based exon excision strategy as a future therapeutic treatment paradigm for human application.

To evaluate whether the CRISPR-Cas9-tool enables the selective excision of *ADGRV1* exons 40–42 in a human genomic context, we employed the HEK293T cell line as a model system. Following an adapted methodology based on Lambrus and colleagues,[19] we generated PX459 CRISPR-Cas9 expression plasmids containing sgRNAs targeting either *ADGRV1* intron 39 or intron 42, which were subsequently transfected into HEK293T cells.

To assess the CRISPR-Cas9-mediated genome editing efficiency, we extracted genomic DNA from transfected cells and performed PCR amplification followed by Sanger sequencing and TIDE analysis.[20] Transfection of the PX459 plasmid carrying an sgRNA targeting intron 39 yielded an average genome editing efficiency of 42.9%, whereas the plasmid with the sgRNA targeting intron 42 achieved an average efficiency of 64.2% (Figure 8A). Given that both sgRNAs enabled potent genome-editing activity, we next co-transfected both plasmids to induce the excision of exons 40–42. Genomic PCR and Sanger sequencing confirmed successful removal of the targeted exons (Figures 8B and 8C).

DISCUSSION

Given the success of (multi-)exon skipping for the future treatment of *USH2A*-associated retinal dysfunction and the current lack of treatment options for *ADGRV1*-associated RP, this study aimed to explore whether a similar exon removal strategy—through CRISPR-Cas9-mediated exon excision—could also serve as a therapeutic strategy for this disorder. Based on the presence of multiple loss-of-function mutations and careful *in silico* protein domain analyses, we selected *ADGRV1* exon 9 and *ADGRV1* exons 40–42 as targets for exon excision. To assess the functional consequences of removing these exons, two zebrafish models were generated in which

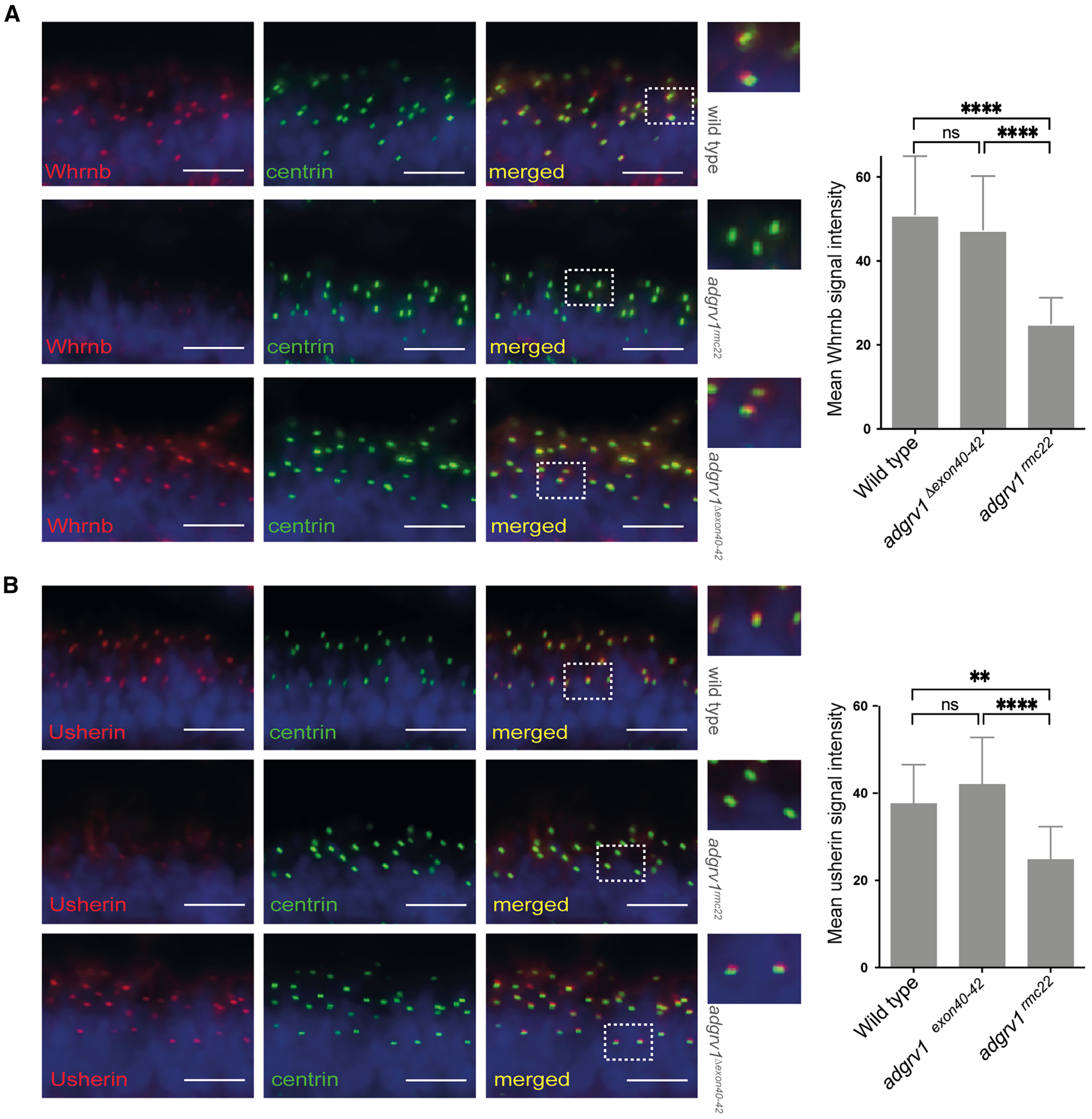

Figure 5. Analysis of usherin and Whrnb expression in retinal sections of wild-type, *adgrv1^{rmc22}*, and *adgrv1$^{\Delta exon40\text{-}42}$* zebrafish

Retinal cryosections of wild-type, mutant *adgrv1^{rmc22}*, and *adgrv1$^{\Delta exon40\text{-}42}$* zebrafish larvae (5 dpf) labeled with antibodies directed against Whrnb (red) (A) or usherin (red) (B) and centrin (green). Nuclei are counterstained with DAPI (blue). (A) Both in wild-type and in *adgrv1$^{\Delta exon40\text{-}42}$* zebrafish larvae, Whrnb was present at the photoreceptor periciliary region in close proximity to centrin. Quantification revealed that the Whrnb signal intensity in *adgrv1$^{\Delta exon40\text{-}42}$* retinal sections is similar to that in wild-type sections, whereas a significant reduction of Whrnb signal was observed in retinae of *adgrv1^{rmc22}* larvae. Magnified images are shown at the right side. Bar graphs represent the mean fluorescence intensity of anti-Whrnb staining of all photoreceptors of a single, central section of one larval zebrafish eye (n = 14 eyes). (B) Both in wild-type and *adgrv1$^{\Delta exon40\text{-}42}$* zebrafish larvae, usherin was present at the photoreceptor periciliary region in close proximity to centrin. Quantification reveals that the usherin signal intensity in retinal sections of *adgrv1$^{\Delta exon40\text{-}42}$* larvae is similar to that in sections of wild types, whereas a significant reduction of usherin signal was observed in retinae of *adgrv1^{rmc22}* larvae. Bar graphs represent the mean fluorescence intensity of anti-usherin staining of all photoreceptors of a single, central section of one larval zebrafish eye, with mean ± SD (n = 14 eyes). Data were analyzed using one-way ANOVA followed by Tukey's multiple comparison test (**$p < 0.01$; ****$p < 0.0001$). Scale bars, 10 μm.

the orthologous exons were excised using CRISPR-Cas9 genome editing. Functional analysis of the *adgrv1$^{\Delta exon9}$* and *adgrv1$^{\Delta exon40\text{-}42}$* zebrafish lines revealed that excision of exon 9 failed to result in the expression of detectable Adgrv1 protein levels, whereas excision of exons 40–42 led to the production of a correctly localized Adgrv1$^{\Delta Exon40\text{-}42}$ protein in photoreceptor cells. Notably, the expression and localization of USH2 complex members were preserved in *adgrv1$^{\Delta exon40\text{-}42}$* zebrafish, and the transport and localization of the photopigment rhodopsin as well as the recorded ERG responses were comparable to those of strain- and age-matched wild-type

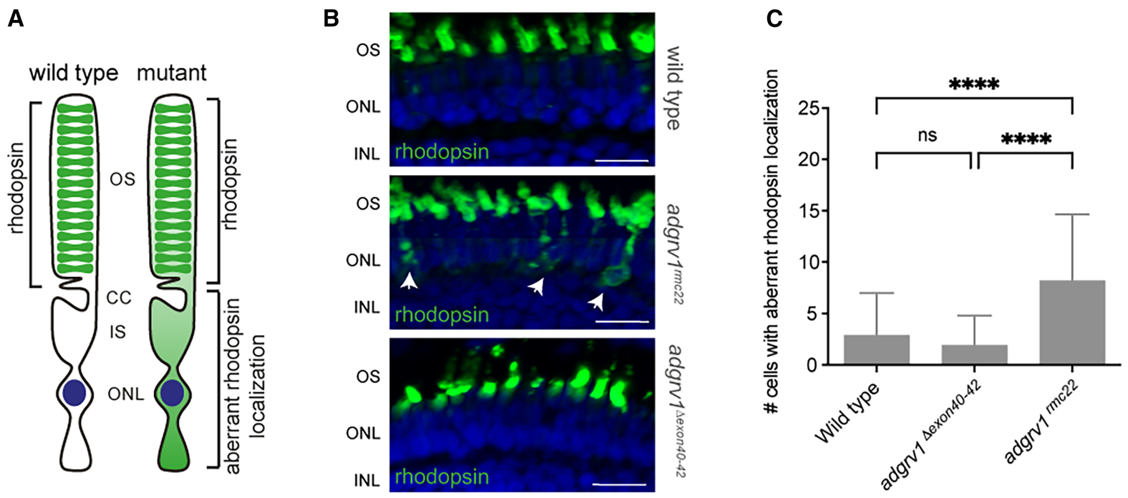

Figure 6. Analysis of rhodopsin localization in retinal sections of wild-type, *adgrv1*^{rmc22}, and *adgrv1*$^{\Delta exon40-42}$ zebrafish

(A) Schematic representation of a photoreceptor with rhodopsin localization in the outer segments (OS) and aberrant rhodopsin localization in the photoreceptor cell body as observed in *adgrv1*^{rmc22} larvae. (B) Retinal cryosections of wild-type, *adgrv1*^{rmc22}, and *adgrv1*$^{\Delta exon40-42}$ zebrafish larvae (6 dpf) labeled with antibodies directed against rhodopsin (green). Nuclei are counterstained with DAPI (blue). As observed in the retinas of wild-type and *adgrv1*$^{\Delta exon40-42}$ zebrafish larvae, rhodopsin predominantly localized to the photoreceptor outer segments. Photoreceptor cells with aberrant localization of rhodopsin were regularly observed in *adgrv1*^{rmc22} larvae (indicated with the white arrows). Scale bars, 10 μm, OS, outer segments; ONL, outer nuclear layer; INL, inner nuclear layer. (C) Total number of cells with aberrant rhodopsin localization per retinal section were plotted, with mean ± SD (n = 41 wild types, n = 42 *adgrv1*$^{\Delta exon40-42}$, and n = 40 *adgrv1*^{rmc22} larvae). Data were analyzed using one-way ANOVA followed by Tukey's multiple comparison test (****$p < 0.0001$).

zebrafish. Finally, we demonstrated that CRISPR-Cas9 genome editing allowed for the successful excision of the genomic region comprising *ADGRV1* exons 40–42 from a human cell line. Our study provides the first evidence that exon excision may represent a viable therapeutic approach for *ADGRV1*-associated RP caused by pathogenic variants in exons 40–42.

Prior to selecting target exons, we conducted a thorough cross-comparison between human and zebrafish ADGRV1 protein structures to identify target exons that would not compromise protein folding and therefore functionality upon removal. Interestingly, 3D models of ADGRV1 revealed a more complex domain architecture compared to previous 2D assessments. AlphaFold2-based models predicted a larger number of Calxβ domains and demonstrated that the laminin G-like domain, cysteine-rich domain, and EAR repeats protrude from—and interrupt—the otherwise continuous Calxβ domain sequence. Since UniProt and SMART 2D models define protein domain boundaries primarily based on amino acid sequence data,[14,21] this may have limited the identification of additional Calxβ domains, potentially explaining the discrepancy with the number of Calxβ domains identified by AlphaFold2. It is important to note, however, that AlphaFold2 models remain predictions that require experimental validation. Limitations include reduced accuracy in modeling intrinsically disordered regions and loop structures, as well as the prediction of static models, whereas proteins are inherently dynamic and can adopt multiple conformational states.[22,23] Nonetheless, given the substantial time and costs associated with experimental modeling approaches such as X-ray crystallography, AlphaFold2 offers a valuable and accessible tool for preliminary structural analyses. In this study, we therefore used AlphaFold2 to assess the structural consequences of exon excision and to guide the selection of therapeutic targets.

Our functional analyses using zebrafish models suggest that the 3D structural predictions by AlphaFold2 offer improved insights into the effects of exon excision compared to SMART-derived 2D protein models. This is demonstrated by the absence of Adgrv1$^{\Delta exon9}$ protein in the retinae of *adgrv1*$^{\Delta exon9}$ zebrafish. The SMART-based prediction of exon 9 excision, which indicated the removal of a single Calxβ domain, may not accurately reflect the true consequences of removing the selected exon on protein domain architecture and structure. More importantly, our study shows that incorporating 3D structural modeling into target selection marks a shift in exon skipping strategy: moving beyond the conventional focus on exons that precisely align with protein domain boundaries.[12] We here provide the first evidence that the removal of exons encoding partial Calxβ domains may result in the production of functional hybrid domains, thereby expanding the range of therapeutically viable exon excision targets. This is exemplified by the ADGRV1$^{\Delta exon40-42}$ protein models: while SMART and AlphaFold2 initially both indicated that these exons encode two partial Calxβ domains—suggesting this region may not be an ideal therapeutic target—AlphaFold2 predictions revealed that upon removal of these exons the remaining counterparts assemble into a novel hybrid Calxβ domain. This hybrid domain exhibits a 3D structure highly similar to native Calxβ domains in both human and zebrafish ADGRV1. The presence and

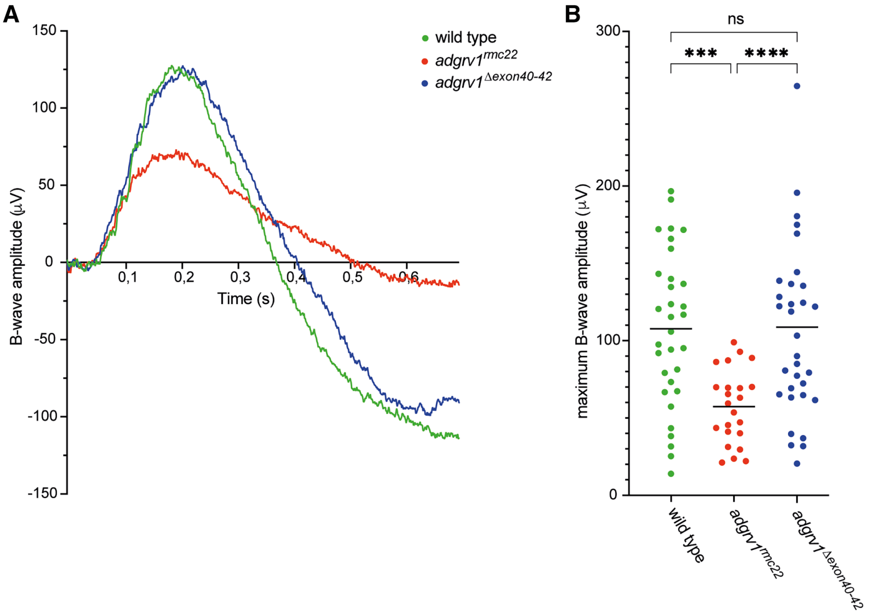

Figure 7. ERG recordings reveal normal retinal function in *adgrv1*$^{\Delta exon40\text{-}42}$ zebrafish

(A) Representative ERG traces of wild-type, *adgrv1*^{rmc22}, and *adgrv1*$^{\Delta exon40\text{-}42}$ zebrafish larvae (7 dpf). (B) Maximum B-wave amplitudes following a light stimulus of 1,000 lux. The mean maximum B-wave amplitude of *adgrv1*$^{\Delta exon40\text{-}42}$ larvae is similar to those observed in wild types, whereas *adgrv1*^{rmc22} larvae show a significant decrease in maximum B-wave amplitudes (***p = 0.0001; ****p < 0.0001; one-way ANOVA followed by Tukey's multiple comparison test).

correct localization of Adgrv1$^{\Delta exon40\text{-}42}$ in zebrafish photoreceptor cells, combined with functional analyses demonstrating that upon genomic excision of exons 40–42 the photoreceptor cell function is preserved, implies that this hybrid Calxβ domain is indeed well tolerated and functional.

Whether this approach can be extended to other Calxβ domains, and which portions of ADGRV1 can be excised while maintaining a functional protein, remains to be determined. This will likely require a better understanding of the role of Calxβ domains. Calxβ domains are named after their homology to regulatory domains in Na^+/Ca^{2+} exchangers.[5] It has been shown that Calxβ domains in ADGRV1 bind Ca^{2+},[24] and due to their abundance in ADGRV1's extracellular region, it has been suggested that the protein may function as an extracellular Ca^{2+} sink.[3] Moreover, it has been hypothesized that these Ca^{2+}-binding domains mediate receptor-ligand interactions[25]; however, to date no specific ligand for ADGRV1 has been identified.

Research on the three USH2-associated proteins—ADGRV1, usherin, and whirlin—has demonstrated that they form a protein complex and are dependent on each other for their proper localization in multiple species and in different organs.[16,17,26] It has been postulated that ADGRV1 and usherin form both homo- and heteromeric dimers that bridge the gap between the membrane of the photoreceptor connecting cilium and the periciliary membrane, although it is yet unclear which regions within the extracellular tails of both proteins are critical for these interactions. PCDH15 serves as a compelling example of how insights into protein domain folding and domains essential for protein-protein interactions can inform therapeutic design: detailed structural and functional analyses of its interaction and dimerization domains[27–29] enabled the identification of the most critical domains to include in the development of effective *PCDH15* minigene therapies.[30] Achieving similar insights for ADGRV1 will be essential to ensure that exon excision preserves both protein folding and its interactions within the USH2 complex. The recent development of AlphaFold3, which enables structural predictions of complex biomolecular interactions,[31] may allow a more comprehensive evaluation of the effects of exon excision on ADGRV1's domain architecture and its interactions with USH2 complex members in the future. Finally, the therapeutic *PCDH15* minigene was about 63% of the size of the full-length gene, indicating that a considerable part of the gene can be missed without disrupting protein function. Similarly, dual-exon skipping studies for the future treatment of *USH2A*-associated RP demonstrated that the deletion of ∼100 amino acids in the extracellular domain yields a shortened yet functional usherin protein, capable of supporting proper localization of USH2 complex members at the periciliary region in zebrafish photoreceptor cells.[12] Notably, this deletion is comparable in size to the 120 amino acids removed by excision of *ADGRV1* exon 40–42.

To facilitate the translation of our findings into a clinical application, several therapeutic approaches may be employed to induce removal of *ADGRV1* exons 40–42. For example, ASOs can be used to transiently modulate splicing by binding complementary to pre-mRNA sequences. This binding either blocks or promotes interactions with the splicing machinery, to induce exon skipping or to promote exon retention. ASO-based therapies are generally considered safe because they target the transcriptome rather than the genome, and intravitreal delivery of naked ASOs has shown limited off-target effects and inflammatory responses in preclinical studies (Trials #NCT03780257 and #NCT03140969). To further enhance their stability, binding affinity, and solubility, various chemical modifications have been developed over the years, including alterations to the backbone, sugar moiety, and 2′-alkyl groups.[32] Furthermore, the therapeutic potential of ASO-induced exon skipping has been well established, with several splice-modulating ASOs that have already received approval from the Food and Drug Administration (FDA). For example, nusinersen—a phosphorothioate (PS) ASO with 2′-O-methoxyethyl (2′-O-MOE) modifications—was designed to

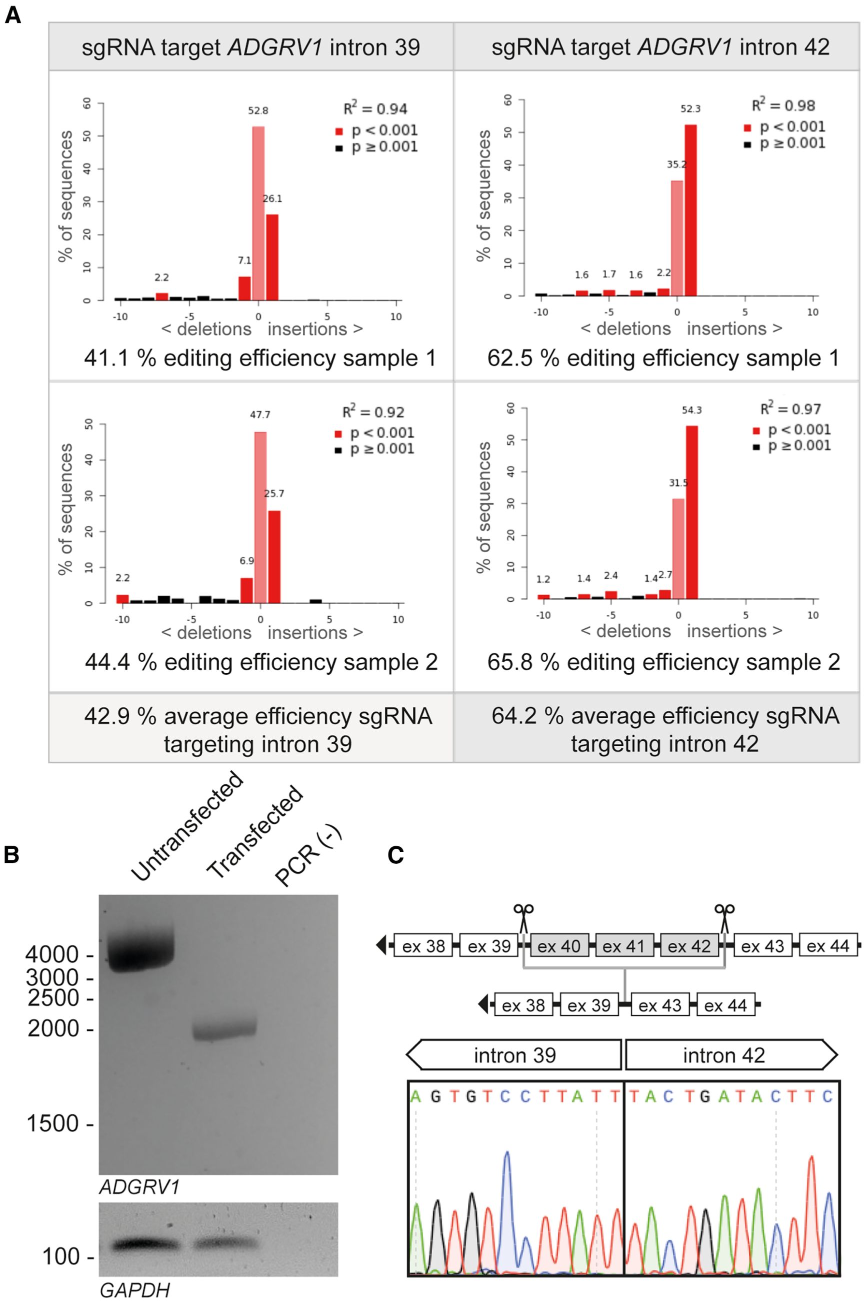

Figure 8. Assessment of CRISPR-Cas9 mediated editing efficiency and successful genomic excision of *ADGRV1* exons 40–42 in HEK293T cells

(A) Assessment of genome-editing efficiency using TIDE analysis[20] following HEK293T transfection with PX459 CRISPR-Cas9 expression plasmids containing sgRNAs targeting either *ADGRV1* intron 39 or intron 42 (n = 2 biological replicates per target). (B) The size of the genomic PCR fragments generated using primers in introns 39 and 42 confirmed the successful excision of *ADGRV1* exons 40–42 from HEK293T cell DNA following co-transfection with both plasmids (unedited amplicon size: 3,997 bp; amplicon size after genomic exon excision: 1,950 bp). GAPDH amplification is shown as a loading control (amplicon size: 110 bp). PCR (–), negative PCR control. (C) Sanger sequencing of amplicons confirmed the successful removal of target exons.

induce exon 7 inclusion in *SMN2* and received FDA approval for the treatment of spinal muscular atrophy.[33] For Duchenne muscular dystrophy, four phosphorodiamidate morpholino oligomer (PMO)-based ASOs (eteplirsen, golodirsen, viltolarsen, and casimersen) designed to induce exon skipping in *DMD*[34–37] have received market approval. Furthermore, ultevursen, a PS-ASO with 2′-O-MOE chemistry designed to promote *USH2A* exon 13 skipping is currently being evaluated in a phase 2b clinical trial (Trial #NCT06627179).[9] However, the simultaneous skipping of multiple exons, such as *ADGRV1* exons 40–42, poses technical difficulties, as it requires concurrent binding of several ASOs to the same pre-mRNA molecule.[38] These challenges have also been observed in *USH2A* dual exon 30–31 and exon 39–40 skipping strategies, where individual ASOs effectively induced skipping of single exons, but their combination led to only a small fraction of correctly spliced transcripts.[12] A likely explanation is that upon delivery, ASOs may not be uniformly taken up by all target cells, limiting the efficiency of multi-exon skipping. A potential solution may be the use of viral vectors to deliver antisense sequences encoded within modified U7 small nuclear RNAs (U7snRNAs).[39] This enables sustained, endogenous production of ASO sequences within transduced cells, ensuring that all required splice-modulating sequences are present simultaneously. Supporting this approach, Goyenvalle and colleagues[40] showed that AAV-delivered U7snRNA constructs can efficiently induce multi-exon skipping in *DMD* with stable, long-term expression, even when targeting up to three exons simultaneously. Although no U7snRNA-based therapies have yet received market approval, a first phase 1/2 clinical trial is currently underway evaluating AAV-delivered U7snRNA-mediated exon skipping for *DMD* (Trial #NCT04240314). This approach may therefore offer a promising strategy for achieving simultaneous skipping of *ADGRV1* exons 40–42.

An alternative strategy is the permanent excision of target exons from the genome using CRISPR-Cas9-based DNA editing technology. In this study, we provided the first evidence that the CRISPR-Cas9 system enables the selective removal of *ADGRV1*

exons 40–42 from the human genome, as demonstrated by successful excision in HEK293T cells. To further explore the therapeutic potential of this approach, follow-up studies are needed in cellular models with more relevant genetic and transcriptional profiles, such as patient-derived 3D retinal organoids. However, recent clinical advancements support the feasibility of CRISPR-based therapeutic approaches. As such, the first FDA approval for a CRISPR-Cas9 therapy was granted for the treatment of sickle cell dise.[41] For retinal applications, a phase 1/2 clinical trial evaluated the safety and efficacy of EDIT-101, a CRISPR-Cas9-based therapy for the excision of the pathogenic c.2991+1655A>G variant in *CEP290* causing Leber congenital amaurosis[42] (Trial #NCT03872479). While the treatment was proven to be safe, its therapeutic efficacy was limited: only patients homozygous for the pathogenic variant showed clinically meaningful improvement, whereas compound heterozygous individuals showed no benefit. This modest outcome likely reflects insufficient editing efficiency.

To address these efficiency issues, and minimize the risk of off-target edits, several studies have focused on improving the CRISPR editing precision, including the creation self-limiting Cas9 systems, CRISPR interference (CRISPRi) platforms, and high-fidelity Cas9 variants.[43,44] Nevertheless, the use of viral vectors encoding Cas9 and gRNAs, such as used in the EDIT-101 trial, raises concerns about potential immune responses due to sustained expression of bacterial Cas9 protein. To mitigate these risks, an alternative approach involves delivering pre-assembled Cas9-gRNA ribonucleoprotein (RNP) complexes. Because RNPs are transiently present in cells, they significantly reduce the window for off-target effects and immune activation. For the delivery of RNPs, non-viral delivery systems such as nanoparticles are being explored. Various formulations—including lipid-, peptide-, polymer-, and inorganic-based nanoparticles—have been developed to enhance cellular uptake and protect genome-editing components from degradation.[45] However, nanoparticle-based delivery vehicles often face challenges related to toxicity, instability of the delivery complex, and variable editing efficiency.[45] In an effort to address these issues, a recent study by Vázquez-Domínguez and colleagues[46] demonstrated that the amphipathic lipopeptide C18:1-LAH5 enables efficient delivery of RNP complexes to retinal cells *in vitro*. Using this approach, they achieved successful excision of an intronic region of *ABCA4* harboring deep intronic pathogenic variants causing Stargardt disease. While promising, these results have so far only been validated in cellular models, highlighting the need for *in vivo* studies to establish clinical relevance in the retina.

In conclusion, our study demonstrates that excision *ADGRV1* exons 40–42 represents a promising therapeutic strategy for the future treatment of *ADGRV1*-associated RP. Moreover, by incorporating 3D structural protein modeling to guide the selection of exon excision targets, we provided the first evidence that removal of exons encoding partial domains can still result in the formation of functional hybrid domains. This finding challenges the prevailing notion that only exons encoding complete protein domains are suitable targets for excision, thereby broadening the therapeutic potential of this approach—not only for *ADGRV1* but also potentially for other genes. Collectively, our work highlights the invaluable role of 3D protein modeling, which not only provided insights into the more complex ADGRV1 protein domain organization than previously understood but also proves instrumental in identifying well-defined targets for personalized exon-excision therapies.

MATERIALS AND METHODS

In silico modeling of 2D and 3D protein domain architectures of human and zebrafish ADGRV1

Amino acid sequences of human and zebrafish ADGRV1 were obtained from the UniProt KB database[21] (UniProt: Q8WXG9 and UniProt: Q6JAN0) and used to predict the 2D protein domain architecture using the SMART online tool.[14] 3D protein structures were modeled with AlphaFold2,[15] using the Google Colab notebook (v1.5.3) with standard settings. Due to the size limitations of AlphaFold2, which precludes modeling the full-length ADGRV1 proteins, the ADGRV1 amino acid sequences were divided into smaller segments, each comprising 800–900 residues, with a minimum overlap of 100 residues between adjacent segments. Next, the predicted 3D structures for each segment were visualized using YASARA[47] and aligned using the MUSTANG algorithm[48] to generate the full-length ADGRV1 models.

In silico modeling of the effect of *ADGRV1* exon 9 and exons 40–42 excision on Calxβ protein domain structures

The impact of removing *ADGRV1* exon 9 and exons 40–42 on the 3D protein structure on both human and zebrafish ADGRV1 proteins was assessed by modeling a segment encoding a chain of four Calxβ domains, using ADGRV1 amino acid sequences UniProt:: Q8WXG9 and UniProt: Q6JAN0. For the wild-type structures, Calxβ domains 3, 4, 5, and 6 were modeled to visualize the protein domains encoded by exons 6–12. Similarly, Calxβ domains 20, 21, 22, and 23 were modeled to examine the protein structures encoded by exons 35–45. The effect of excision of exon 9 or exons 40–42 was then simulated by removing the amino acid sequences encoded by the target exons. 3D protein structures were generated with AlphaFold2,[15] using the Google Colab notebook (v1.5.3) with standard settings. Protein structures were visualized using YASARA.[47] For the structural comparison of the Calxβ domains, native human and zebrafish Calxβ domains 4, 5, 21, and 22, and hybrid Calxβ domains following removal of exon 9 and exons 40–42, were superimposed by aligning these structures using the MUSTANG algorithm.[48]

Zebrafish ethics, maintenance, and husbandry

Animal experiments were conducted in compliance with the Dutch guidelines for laboratory animal care and use (Wet op de Dierproeven 1996), as well as European regulations (Directive 86/609/EED), and approved by the Dutch Ethics Committee of the Central Committee Animal Experimentation (Centrale Commissie Dierproeven [CCD] application number AVD10300 2022 15892). Zebrafish were maintained and raised according to standard protocols.[49] Both larval and adult zebrafish were kept under a 14-h light (300 lux) and 10-h

dark cycle. Adult zebrafish were fed daily with Gemma Micro 300 dry pellets (#13177, Zebcare, Nederweert, the Netherlands) at approximately 5% of their body weight and provided with artemia as a supplemental food source. Embryos were obtained through natural spawning. We utilized wild-type AB-strain zebrafish to generate the *adgrv1*$^{\Delta exon9}$ (c.1486-1020_1815+22delinsGGT; p.M496_Q605del) and *adgrv1*$^{\Delta exon40\text{-}42}$(c.8768-442_9127+1374del; p.D2923_A3043delinsT) therapeutic models, which were deposited as ZFIN: *adgrv1*^{rmc25} and ZFIN: *adgrv1*^{rmc31} in the ZFIN repository, respectively (ENSDART00000008043.9). Additionally, we employed the previously described ZFIN: *adgrv1*^{rmc22} mutant zebrafish.[16]

CRISPR-Cas9 genome-editing design and microinjections

To enable the generation of therapeutic *adgrv1*$^{\Delta exon9}$ and *adgrv1*$^{\Delta exon40\text{-}42}$ zebrafish lines, sgRNAs were designed to target the intronic sequences up- and downstream of *adgrv1* (ENSDART00000008043.9) exon 9 and exons 40–42 using the web tool CRISPRscan.[50] Consequently, sgRNAs were selected with target sites in *adgrv1* introns 8 and 9 and introns 39 and 42, respectively. A cutoff value of at least four mismatches for potential off-target regions was used. Based on the CRISPRscan predictions, Alt-R CRISPR-Cas9 sgRNAs were ordered from Integrated DNA Technologies (IDT) for the indicated intronic target sequences. The genomic target sequences are listed in Table S1.

For the generation of the *adgrv1*$^{\Delta exon9}$ and *adgrv1*$^{\Delta exon40\text{-}42}$ zebrafish, the 5′ sgRNA, 3′ sgRNA, and commercial Alt-R S.p. Cas9 Nuclease V3 (IDT, Coralville, IA, USA, #1081059) were co-injected in wild-type AB-strain zebrafish embryos at the single-cell stage. To avoid preferential *in vivo* binding of Cas9 to either sgRNA, individual sgRNA-Cas9 complexes were prepared and incubated at 37°C for 5 min to allow sgRNA-Cas9 ribonucleoprotein complex formation. Subsequently, for the generation of the *adgrv1*$^{\Delta exon9}$ line, these mixtures were combined to obtain the final injection mixture that consisted of 100 ng/μL of 5′ sgRNA, 100 ng/μL of 3′ sgRNA, 800 ng/μL Cas9 Nuclease, 300 mM KCl, and 20% (v/v) phenol red solution (#P0290, Sigma Aldrich, Amsterdam, the Netherlands). To generate the *adgrv1*$^{\Delta exon40\text{-}42}$ line, a similar injection mixture was prepared, but with an adjustment to use 200 ng/μL of the 3′ sgRNA in order to achieve optimal editing efficiency; 1 nL of this mixture was injected into single-cell-stage zebrafish embryos using a Pneumatic PicoPump pv280 (World Precision Instruments, Friedberg, Germany). Following the injection, the embryos were raised at 28.5°C in E3 embryo medium consisting of 5 mM NaCl, 0.17 mM KCl, 0.33 mM CaCl2, 0.33 mM MgSO4, and 0.1% (v/v) methylene blue. At 1 dpf, 16 out of 100 injected embryos were analyzed for the presence of the anticipated deletions using genomic PCR analysis. The remainder of the injected embryos were raised to adulthood. Founder fish that transmitted anticipated germline lesions to their progeny were outcrossed with wild-type zebrafish for two generations to further minimize the possible presence of unforeseen CRISPR-Cas9-induced off-target modifications. After the second round of outcrossing, heterozygous fish were inbred to produce progeny, of which the homozygous fish were used for subsequent breeding and phenotypic analysis.

Genotyping

Genomic DNA was isolated from whole larvae (1 dpf) or caudal fin tissue from adult zebrafish. Tissues were lysed in 25 μL (larvae) or 75 μL (fin tissue) hotshot lysis buffer (40 mM NaOH and 0.2 mM EDTA (Invitrogen, Waltham, MA, USA, #15575-038) at 95°C for 20 min and subsequently neutralized by adding 7.5 μL 1M Tris-HCl pH 7.5. Lysates were diluted 10x with MilliQ, and 1 μL of the diluted sample was used as template for the PCR. To amplify the *adgrv1*$^{\Delta exon9}$ zebrafish allele, and the corresponding wild-type *adgrv1* allele, a PCR analysis was performed using the Taq DNA polymerase (Roche, Basel, Switzerland, #11647687001). A Q5 High-Fidelity DNA Polymerase Kit (New England Biolabs, Ipswich, MA, USA, #M0491L) was used for the amplification of the *adgrv1*$^{\Delta exon40\text{-}42}$ zebrafish allele and the corresponding wild-type *adgrv1* allele. Afterward, amplicons were size-separated on a 2% agarose gel. Sanger sequencing of PCR amplicons confirmed the correct removal of the target exons. All primer sequences are listed in Table S2.

Transcript analysis

To enable transcript analysis, total RNA was isolated from three pooled larvae (5 dpf) of each genotype: *adgrv1*$^{\Delta exon9}$, *adgrv1*$^{\Delta exon40\text{-}42}$, and wild-type AB. The larvae were homogenized prior to lysis, and total RNA was extracted using the RNeasy Micro kit in accordance with the manufacturer's guidelines (Qiagen, Hilden, Germany, #74004). Subsequently, 250 μg RNA was used for cDNA synthesis using the SuperscriptTM IV Reverse Transcriptase kit (Thermo Fisher Scientific, Waltham, MA, USA, #18090200). For *adgrv1*$^{\Delta exon9}$ transcript analysis, a segment spanning from exon 7 to exon 11 (1,500 bp for wild-type transcripts and 1,140 bp for *adgrv1*$^{\Delta exon9}$ transcripts) was amplified using Q5 High-Fidelity DNA Polymerase (New England Biolabs, Ipswich, MA, USA, #M0491L). Similarly, to enable *adgrv1*$^{\Delta exon40\text{-}42}$ transcript analysis, an amplicon spanning from exon 35 to exon 45 (1,463 bp for wild-type transcripts, 1,103 bp for *adgrv1*$^{\Delta exon40\text{-}42}$ transcripts) was generated using the Q5 High-Fidelity DNA Polymerase kit. Afterward, amplicons were size-separated on a 2% agarose gel and analyzed using Sanger Sequencing. All primer sequences are listed in Table S2.

Immunohistochemistry, histology, and quantification of fluorescent signal intensity

Larvae (5 dpf) of homozygous *adgrv1*$^{\Delta exon9}$, *adgrv1*$^{\Delta exon40\text{-}42}$, *adgrv1*^{rmc22} and wild-type AB strain zebrafish were cryoprotected by a 5-min incubation in 10% sucrose in PBS prior to embedding in OCT compound (Sakura, Alphen aan den Rijn, the Netherlands, Tissue-Tek, #4583). After the embedding procedure, the samples were slowly frozen using liquid-nitrogen-cooled isopentane, and standard protocols were followed to prepare cryosections of 7 μm. Unfixed cryosections were permeabilized for 20 min using a 0.01% Tween 20 solution in PBS, followed by a 30-min incubation with blocking buffer. For immunohistochemical analysis of Adgrv1, a blocking buffer of 10% normal goat serum and 2% bovine serum albumin in PBS was used. For Whrnb and usherin, a blocking buffer of 10% non-fat dry milk in PBS was used. Primary and secondary antibodies were diluted in the corresponding blocking buffers. Cryosections were incubated

overnight at 4°C with the primary antibodies, followed by three 10-min rinses with PBS. Sections were then incubated for 1 h with the secondary antibodies along with DAPI (1:800; D1306; Thermo Fisher, Waltham, MA, USA). Following a second round of rinsing (three times for 10 min with PBS), cryosections were post-fixed using 4% paraformaldehyde for 10 min, followed by rinsing three times for 5 min with PBS. Finally, Prolong Gold Anti-fade (P36930; Thermo Fisher, Waltham, MA, USA) was used for mounting the cryosections. The following primary antibodies and dilutions were used: rabbit anti-usherin (1:1000; #DZ01481, Boster Bio, Pleasanton, CA, USA), rabbit anti-Adgrv1 (1:100; #DZ41032; Boster Bio), rabbit anti-Whrnb (1:750; #42690002, Cip98a; Novus Biologicals, Centennial, CO, USA), and mouse anti-centrin (1:500; #04–1624; Millipore, Darmstadt, Germany). Secondary antibodies Alexa Fluor 568 goat anti-rabbit (#A-11011, Thermo Fisher, Waltham, MA, USA) and Alexa Fluor 488 goat anti-mouse (#A-11029, Thermo Fisher, Waltham, MA, USA) were used in a 1:800 dilution. Fluorescence microscopy imaging of the slides was performed using a Zeiss Axio Imager microscope equipped with an AxioCam MCR5 camera (Zeiss, Jena, Germany). The intensity of the usherin and Whrnb immunofluorescence was quantified using an automated Fiji (v.1.51n) script as previously published.[16]

For the rhodopsin immunolabeling, *adgrv1*$^{\Delta exon40\text{-}42}$, *adgrv1*^{rmc22}, and wild-type AB zebrafish larvae were raised in transparent 10-cm Petri dishes under standard light conditions (300 lux white light in a 14-h day, 10-h night cycle). The larvae were sampled at 6 dpf precisely 100 min after light onset. Coronal cryosections (7 μm) of 4% PFA fixed larvae were taken from the central retina at the level of the optic nerve, with one section per larva used for analysis. Sections were stained and imaged as published previously.[51] Rhodopsin was immunolabeled using a primary mouse anti-rhodopsin antibody (1:4,000, Clone 4D2; NBP2-59690, Novus Biologicals, Centennial, CO, USA) and a secondary Alexa Fluor 488 goat anti-mouse antibody (Thermo Fisher, Waltham, MA, USA, #A-11029) combined with DAPI (1:8,000; D1306; Thermo Fisher, Waltham, MA, USA). Fluorescence microscopy imaging of the slides was performed using a Zeiss Axio Imager microscope equipped with an AxioCam MCR5 camera (Zeiss, Jena, Germany). Subsequently, two independent researchers quantified rhodopsin mislocalization in a blinded and randomized manner, by analyzing the full photoreceptor cell layer of each section and manually scoring the number of photoreceptor cells exhibiting a clear rhodopsin signal within the cell body. In total n = 41 wild-type sections, n = 42 *adgrv1*$^{\Delta exon40\text{-}42}$ sections, and n = 40 *adgrv1*^{rmc22} sections were analyzed.

Electroretinogram recordings

Electroretinogram (ERG) recordings were conducted on isolated eyes of zebrafish larvae (7 dpf). The larvae were raised under bright light conditions at 3000 lux from 1 dpf to 6 dpf, with a 12-h light and 12-h dark cycle. On the morning of 7 dpf, the larvae were kept in the dark and handled under dim red light until the ERG recordings were performed on the isolated eyes, as previously described.[17] The recording protocol included the following adaptations: after positioning the electrode, a 3-min waiting period was introduced, followed by three light pulses (at 100 lux, 500 lux and 1000 lux) with a 1-min interval between the different light intensities. Each recording lasted 2 s, with a 50-millisecond light pulse, and the average maximal B-wave amplitude was plotted.

Exon excision in HEK293T cells

To enable the excision of the genomic region of *ADGRV1* (ENST00000405460.9) containing exons 40–42 in HEK293T cells, sgRNAs were designed to target introns 39 and 42, using the web tool CRISPRscan.[50] CRISPRscan was used to search for potential genomic off-targets with up to four mismatches. Based on the predictions, the sgRNA sequences listed in Table S1 were selected, as they were predicted to have no off-target sites.

CRISPR-Cas9-mediated genome editing was performed following the method described by Lambrus and colleagues,[19] using the PX459 CRISPR-Cas9 expression plasmid that uses a U6 promoter to transcribe the specific sgRNAs. To generate the PX459 plasmids containing the sgRNA sequences, the PX459 plasmid (Addgene #101715) was first linearized with BbsI. Next, the oligonucleotides that were designed to enable a directional integration of sgRNA sequences (Table S3) were annealed, creating an overhang compatible with the BbsI site, and subsequently ligated into the linearized PX459 vector using T4 DNA ligase (#M1801, Promega, Madison, Wisconsin, USA), followed by transformation into DH5α *Escherichia coli* cells. After plasmid isolation, correct integration of sgRNA sequences was confirmed by Sanger sequencing.

HEK293T cells were cultured as previously described[12] and seeded in 12-well plates in 1mL Dulbecco's modified Eagle's medium (DMEM; #D0819, Sigma-Aldrich, St. Louis, MO, USA) supplemented with 10% (v/v) fetal bovine serum (FBS; #F7524, Sigma-Aldrich, St. Louis, MO, USA), 1% penicillin-streptomycin (#P4333, Sigma-Aldrich, St. Louis, MO, USA), and 1% sodium pyruvate (#S8636, Sigma-Aldrich, St. Louis, MO, USA). After 24 h of incubation at 37°C, when the cells reached approximately 70% confluency, transfection was performed using 90 μL of polyethylenimine (PEI) combined with 500 μg plasmid DNA of either a single PX459 plasmid or a combination of two PX459 plasmids containing sgRNAs targeting intron 39 and intron 42.

At 48 h post-transfection, cells were washed with 1x PBS, trypsinized, and split at a ratio 1:2 and seeded in new 12-well plates in 1mL DMEM. Once cells reattached (∼52 h post-transfection), the medium was replaced with DMEM containing 1 μg/mL puromycin (#P8833, Sigma-Aldrich, St. Louis, MO, USA) to select for successfully transfected cells. After 48 h of puromycin selection, the medium was replaced to DMEM medium without puromycin. At 24 h post-puromycin removal (day 6 post-transfection), cells were harvested.

Genomic DNA was extracted from cell pellets by incubating samples at 95°C for 20 min in 200 μL hotshot lysis buffer (40 mM NaOH and 0.2 mM EDTA [Invitrogen, Waltham, MA, USA, #15575-038]). Lysates were neutralized with 20 μL of 1M Tris-HCl (pH 7.5) and

subsequently diluted 10x with MilliQ water. One microliter of the diluted lysate was used as template for PCR. The CRISPR editing efficiency was assessed by amplifying the genomic DNA of cells transfected with a single PX459 plasmid carrying an sgRNA targeting either *ADGRV1* intron 39 or intron 42, using Taq polymerase (# 11647687001, Roche, Basel, Switzerland) and the primers listed in Table S3. PCR products were submitted for Sanger sequencing, and editing efficiency was determined using TIDE analysis, which calculates the frequency of CRISPR-induced indels and provides a detailed distribution of indel sizes.[20] For cells transfected with a combination of two PX459 plasmids containing sgRNAs targeting both intron 39 and intron 42, an amplicon spanning intron 39 to intron 42 was generated using Q5 High-Fidelity DNA Polymerase (#M0491L, New England Biolabs, Ipswich, MA, USA). Primers amplifying a genomic segment of *GAPDH* were included as a loading control. All primers are listed in Table S3. PCR products were size-separated on a 2% agarose gel. Sanger sequencing confirmed the correct removal of the target exons.

DATA AVAILABILITY

The datasets analyzed during the present study are available from the corresponding author upon reasonable request.

ACKNOWLEDGMENTS

The authors gratefully acknowledge the Radboud University Zebrafish Facility (www.ru.nl/zebrafish) for their excellent fish husbandry. This study was financially supported by Stichting UitZicht (2019-16), CUREUsher, and Stichting Ushersyndroom.

AUTHOR CONTRIBUTIONS

M.S., investigation, visualization, methodology, project administration, and writing—original draft. L.M., formal analysis, data curation, and writing—review and editing. S. B., investigation, methodology, and writing—review and editing. T.P., investigation and writing—review and editing. I.E., investigation and writing—review and editing. M.I., methodology and writing—review and editing. H.V., data curation and writing—review and editing. H.K., funding acquisition, supervision, and writing—review and editing. E.V., funding acquisition, conceptualization, supervision, and writing—review and editing. E.W., funding acquisition, conceptualization, supervision, and writing—review and editing.

DECLARATION OF INTERESTS

The authors have no competing interest.

SUPPLEMENTAL INFORMATION

Supplemental information can be found online at https://doi.org/10.1016/j.omtn.2025.102702.

REFERENCES

1. Nisenbaum, E., Thielhelm, T.P., Nourbakhsh, A., Yan, D., Blanton, S.H., Shu, Y., Koehler, K.R., El-Amraoui, A., Chen, Z., Lam, B.L., and Liu, X. (2022). Review of Genotype-Phenotype Correlations in Usher Syndrome. Ear Hear. *43*, 1–8.
2. Velde, H.M., Reurink, J., Held, S., Li, C.H.Z., Yzer, S., Oostrik, J., Weeda, J., Haer-Wigman, L., Yntema, H.G., Roosing, S., et al. (2022). Usher syndrome type IV: clinically and molecularly confirmed by novel ARSG variants. Hum. Genet. *141*, 1723–1738.
3. Weston, M.D., Luijendijk, M.W.J., Humphrey, K.D., Möller, C., and Kimberling, W. J. (2004). Mutations in the VLGR1 gene implicate G-protein signaling in the pathogenesis of Usher syndrome type II. Am. J. Hum. Genet. *74*, 357–366.
4. Breazzano, M.P., Grewal, M.R., Tsang, S.H., and Chen, R.W.S. (2023). Etiology of Retinitis Pigmentosa. Methods Mol. Biol. *2560*, 15–30.
5. McMillan, D.R., Kayes-Wandover, K.M., Richardson, J.A., and White, P.C. (2002). Very large G protein-coupled receptor-1, the largest known cell surface protein, is highly expressed in the developing central nervous system. J. Biol. Chem. *277*, 785–792.
6. Maerker, T., van Wijk, E., Overlack, N., Kersten, F.F.J., McGee, J., Goldmann, T., Sehn, E., Roepman, R., Walsh, E.J., Kremer, H., and Wolfrum, U. (2008). A novel Usher protein network at the periciliary reloading point between molecular transport machineries in vertebrate photoreceptor cells. Hum. Mol. Genet. *17*, 71–86.
7. Maddalena, A., Tornabene, P., Tiberi, P., Minopoli, R., Manfredi, A., Mutarelli, M., Rossi, S., Simonelli, F., Naggert, J.K., Cacchiarelli, D., and Auricchio, A. (2018). Triple Vectors Expand AAV Transfer Capacity in the Retina. Mol. Ther. *26*, 524–541.
8. Kumaran, N., Michaelides, M., Smith, A.J., Ali, R.R., and Bainbridge, J.W.B. (2018). Retinal gene therapy. Br. Med. Bull. *126*, 13–25.
9. Dulla, K., Slijkerman, R., van Diepen, H.C., Albert, S., Dona, M., Beumer, W., Turunen, J.J., Chan, H.L., Schulkens, I.A., and Vorthoren, L. (2021). Antisense oligonucleotide-based treatment of retinitis pigmentosa caused by USH2A exon 13 mutations. Mol. Ther. *29*, 2441.
10. Aartsma-Rus, A., Janson, A.A.M., Kaman, W.E., Bremmer-Bout, M., van Ommen, G.J.B., den Dunnen, J.T., and van Deutekom, J.C.T. (2004). Antisense-induced multiexon skipping for Duchenne muscular dystrophy makes more sense. Am. J. Hum. Genet. *74*, 83–92.
11. Rutten, J.W., Dauwerse, H.G., Peters, D.J.M., Goldfarb, A., Venselaar, H., Haffner, C., van Ommen, G.J.B., Aartsma-Rus, A.M., and Lesnik Oberstein, S.A.J. (2016). Therapeutic NOTCH3 cysteine correction in CADASIL using exon skipping: *in vitro* proof of concept. Brain *139*, 1123–1135.
12. Schellens, R.T.W., Broekman, S., Peters, T., Graave, P., Malinar, L., Venselaar, H., Kremer, H., De Vrieze, E., and Van Wijk, E. (2023). A protein domain-oriented approach to expand the opportunities of therapeutic exon skipping for USH2A-associated retinitis pigmentosa. Mol. Ther. Nucleic Acids *32*, 980–994.
13. Lee, J.J.A., Maruyama, R., Duddy, W., Sakurai, H., and Yokota, T. (2018). Identification of Novel Antisense-Mediated Exon Skipping Targets in DYSF for Therapeutic Treatment of Dysferlinopathy. Mol. Ther. Nucleic Acids *13*, 596–604.
14. Letunic, I., Khedkar, S., and Bork, P. (2021). SMART: recent updates, new developments and status in 2020. Nucleic Acids Res. *49*, D458–D460.
15. Jumper, J., Evans, R., Pritzel, A., Green, T., Figurnov, M., Ronneberger, O., Tunyasuvunakool, K., Bates, R., Žídek, A., Potapenko, A., et al. (2021). Highly accurate protein structure prediction with AlphaFold. Nature *596*, 583–589.
16. Stemerdink, M., Broekman, S., Peters, T., Kremer, H., de Vrieze, E., and van Wijk, E. (2023). Generation and Characterization of a Zebrafish Model for ADGRV1-Associated Retinal Dysfunction Using CRISPR/Cas9 Genome Editing Technology. Cells *12*, 1598.
17. Dona, M., Slijkerman, R., Lerner, K., Broekman, S., Wegner, J., Howat, T., Peters, T., Hetterschijt, L., Boon, N., de Vrieze, E., et al. (2018). Usherin defects lead to early-onset retinal dysfunction in zebrafish. Exp. Eye Res. *173*, 148–159.
18. Toms, M., Dubis, A.M., de Vrieze, E., Tracey-White, D., Mitsios, A., Hayes, M., Broekman, S., Baxendale, S., Utoomprurkporn, N., Bamiou, D., et al. (2020). Clinical and preclinical therapeutic outcome metrics for USH2A-related disease. Hum. Mol. Genet. *29*, 1882–1899.
19. Lambrus, B.G., Moyer, T.C., and Holland, A.J. (2018). Applying the auxin-inducible degradation system for rapid protein depletion in mammalian cells. Methods Cell Biol. *144*, 107–135.
20. Brinkman, E.K., Chen, T., Amendola, M., and van Steensel, B. (2014). Easy quantitative assessment of genome editing by sequence trace decomposition. Nucleic Acids Res. *42*, e168.
21. UniProt Consortium (2025). UniProt: the Universal Protein Knowledgebase in 2025. Nucleic Acids Res. *53*, D609–D617.
22. Yang, Z., Zeng, X., Zhao, Y., and Chen, R. (2023). AlphaFold2 and its applications in the fields of biology and medicine. Signal Transduct. Target. Ther. *8*, 115.
23. He, X.-H., You, C.-Z., Jiang, H.-L., Jiang, Y., Xu, H.E., and Cheng, X. (2023). AlphaFold2 versus experimental structures: evaluation on G protein-coupled receptors. Acta Pharmacol. Sin. *44*, 1–7.

24. Nikkila, H., McMillan, D.R., Nunez, B.S., Pascoe, L., Curnow, K.M., and White, P.C. (2000). Sequence similarities between a novel putative G protein-coupled receptor and Na+/Ca2+ exchangers define a cation binding domain. Mol. Endocrinol. *14*, 1351–1364.

25. McMillan, D.R., and White, P.C. (2010). Studies on the very large G protein-coupled receptor: from initial discovery to determining its role in sensorineural deafness in higher animals. Adv. Exp. Med. Biol. *706*, 76–86.

26. Yang, J., Liu, X., Zhao, Y., Adamian, M., Pawlyk, B., Sun, X., McMillan, D.R., Liberman, M.C., and Li, T. (2010). Ablation of whirlin long isoform disrupts the USH2 protein complex and causes vision and hearing loss. PLoS Genet. *6*, e1000955.

27. Araya-Secchi, R., Neel, B.L., and Sotomayor, M. (2016). An elastic element in the protocadherin-15 tip link of the inner ear. Nat. Commun. *7*, 13458.

28. De-la-Torre, P., Choudhary, D., Araya-Secchi, R., Narui, Y., and Sotomayor, M. (2018). A Mechanically Weak Extracellular Membrane-Adjacent Domain Induces Dimerization of Protocadherin-15. Biophys. J. *115*, 2368–2385.

29. Sotomayor, M., Weihofen, W.A., Gaudet, R., and Corey, D.P. (2012). Structure of a force-conveying cadherin bond essential for inner-ear mechanotransduction. Nature *492*, 128–132.

30. Ivanchenko, M.V., Hathaway, D.M., Klein, A.J., Pan, B., Strelkova, O., De-la-Torre, P., Wu, X., Peters, C.W., Mulhall, E.M., Booth, K.T., et al. (2023). Mini-PCDH15 gene therapy rescues hearing in a mouse model of Usher syndrome type 1F. Nat. Commun. *14*, 2400.

31. Abramson, J., Adler, J., Dunger, J., Evans, R., Green, T., Pritzel, A., Ronneberger, O., Willmore, L., Ballard, A.J., Bambrick, J., et al. (2024). Accurate structure prediction of biomolecular interactions with AlphaFold 3. Nature *630*, 493–500.

32. Khvorova, A., and Watts, J.K. (2017). The chemical evolution of oligonucleotide therapies of clinical utility. Nat. Biotechnol. *35*, 238–248.

33. Hoy, S.M. (2017). Nusinersen: First Global Approval. Drugs *77*, 473–479.

34. Syed, Y.Y. (2016). Eteplirsen: first global approval. Drugs *76*, 1699–1704.

35. Heo, Y.A. (2020). Golodirsen: First Approval. Drugs *80*, 329–333.

36. Dhillon, S. (2020). Viltolarsen: First Approval. Drugs *80*, 1027–1031.

37. Shirley, M. (2021). Casimersen: First Approval. Drugs *81*, 875–879.

38. Echigoya, Y., Lim, K.R.Q., Nakamura, A., and Yokota, T. (2018). Multiple Exon Skipping in the Duchenne Muscular Dystrophy Hot Spots: Prospects and Challenges. J. Pers. Med. *8*, 41.

39. De Angelis, F.G., Sthandier, O., Berarducci, B., Toso, S., Galluzzi, G., Ricci, E., Cossu, G., and Bozzoni, I. (2002). Chimeric snRNA molecules carrying antisense sequences against the splice junctions of exon 51 of the dystrophin pre-mRNA induce exon skipping and restoration of a dystrophin synthesis in Δ48-50 DMD cells. Proc. Natl. Acad. Sci. USA *99*, 9456–9461.

40. Goyenvalle, A., Wright, J., Babbs, A., Wilkins, V., Garcia, L., and Davies, K.E. (2012). Engineering multiple U7snRNA constructs to induce single and multiexon-skipping for Duchenne muscular dystrophy. Mol. Ther. *20*, 1212–1221.

41. Philippidis, A. (2024). CASGEVY Makes History as FDA Approves First CRISPR/Cas9 Genome Edited Therapy. Hum. Gene. Ther. *35*, 1–4.

42. Pierce, E.A., Aleman, T.S., Jayasundera, K.T., Ashimatey, B.S., Kim, K., Rashid, A., Jaskolka, M.C., Myers, R.L., Lam, B.L., Bailey, S.T., et al. (2024). Gene Editing for CEP290-Associated Retinal Degeneration. N. Engl. J. Med. *390*, 1972–1984.

43. Aquino-Jarquin, G. (2021). Current advances in overcoming obstacles of CRISPR/Cas9 off-target genome editing. Mol. Genet. Metab. *134*, 77–86.

44. Xue, K., and MacLaren, R.E. (2020). Correcting visual loss by genetics and prosthetics. Curr. Opin. Physiol. *16*, 1–7.

45. Kazemian, P., Yu, S.-Y., Thomson, S.B., Birkenshaw, A., Leavitt, B.R., and Ross, C.J. D. (2022). Lipid-nanoparticle-based delivery of CRISPR/Cas9 genome-editing components. Mol. Pharm. *19*, 1669–1686.

46. Vázquez-Domínguez, I., Öktem, M., Winkelaar, F.A., Nguyen, T.H., Hoogendoorn, A.D., Roschi, E., Astuti, G.D., Timmermans, R., Suárez-Herrera, N., and Bruno, I. (2024). Lipopeptide-mediated Cas9 RNP delivery: A promising broad therapeutic strategy for safely removing deep-intronic variants in ABCA4. Mol. Ther. Nucleic Acids *35*, 102345.

47. Krieger, E., and Vriend, G. (2014). YASARA View - molecular graphics for all devices - from smartphones to workstations. Bioinformatics *30*, 2981–2982.

48. Konagurthu, A.S., Whisstock, J.C., Stuckey, P.J., and Lesk, A.M. (2006). MUSTANG: a multiple structural alignment algorithm. Proteins *64*, 559–574.

49. Westerfield, M. (2007). The Zebrafish Book: A Guide for the Laboratory Use of Zebrafish (Danio Rerio) (University of Oregon Press).

50. Moreno-Mateos, M.A., Vejnar, C.E., Beaudoin, J.-D., Fernandez, J.P., Mis, E.K., Khokha, M.K., and Giraldez, A.J. (2015). CRISPRscan: designing highly efficient sgRNAs for CRISPR-Cas9 targeting *in vivo*. Nat. Methods *12*, 982–988.

51. Corral-Serrano, J.C., Messchaert, M., Dona, M., Peters, T.A., Kamminga, L.M., van Wijk, E., and Collin, R.W.J. (2018). C2orf71a/pcare1 is important for photoreceptor outer segment morphogenesis and visual function in zebrafish. Sci. Rep. *8*, 9675.

OMTN, Volume *36*

Supplemental information

Exploring exon excision as a therapeutic intervention strategy for the future treatment of *ADGRV1*-associated retinitis pigmentosa

Merel Stemerdink, Lucija Malinar, Sanne Broekman, Theo Peters, Iris Ensink, Maryna V. Ivanchenko, Hanka Venselaar, Hannie Kremer, Erik de Vrieze, and Erwin van Wijk

SUPPLEMENTAL MATERIAL

Table S1: sgRNA target sequences

**sgRNA target:**		**Sequence (5' → 3')**
Zebrafish *adgrv1* (ENSDART00000008043.9)	intron 8	GGACTTTGCTTGTACCACAG
Zebrafish *adgrv1* (ENSDART00000008043.9)	intron 9	GGTAAACTGTTCAAACCATA
Zebrafish *adgrv1* (ENSDART00000008043.9)	intron 39	AGCGAGGGAAGTGGAAATTG
Zebrafish *adgrv1* (ENSDART00000008043.9)	intron 42	GTCAGAGGTGATTCAGAAAA
Human *ADGRV1* (ENST00000405460.9)	intron 39	ATGCTTATCACGTCTGAATA
Human *ADGRV1* (ENST00000405460.9)	intron 42	CAGTATACCTCAACACTATG

Table S2: Primers used for genomic PCR and RT-PCR analysis of zebrafish samples

	**Primer name**	**Primer sequence (5' → 3')**
***adgrv1$^{\Delta exon9}$* genotyping**	*adgrv1* wild type forward	TATGGTCGATTTGGCTTTCATCC
	adgrv1 wild type reverse*	TGTGTTGTCCCAACTGTAATGTC
	adgrv1$^{\Delta exon9}$ forward	GTTTTGTGGTCCAGGGTCAC
	adgrv1$^{\Delta exon9}$ reverse*	TGTGTTGTCCCAACTGTAATGTC
***adgrv1$^{\Delta exon40-42}$* genotyping**	*adgrv1$^{\Delta exon40-42}$* forward	CAGAAAGCAGGCTATGTC
	adgrv1$^{\Delta exon40-42}$ reverse	CATGCCCATCGTCATTAG
***adgrv1$^{\Delta exon9}$* transcript analysis**	*adgrv1* exon 7-11 forward	GTGCAGATCAAGATCTCCCGT
	adgrv1 exon 7-11 reverse	GTTGTCCACATTTGTCCTGTCT
***adgrv1$^{\Delta exon40-42}$* transcript analysis**	*adgrv1* exon 35-45 forward	GACGGCTCCTGCTATTGGAA
	adgrv1 exon 35-45 reverse	CCTCCACTGTCATGGTGTCC

*Primers are identical

Table S3: Primers used for generation PX459 plasmid and genomic PCR on HEK293T samples

	Primer name	Primer sequence (5' → 3')
**sgRNA integration in PX459**	PX459 *ADGRV1* intron 39 forward	CACCGATGCTTATCACGTCTGAATA
	PX459 *ADGRV1* intron 39 reverse	AAACTATTCAGACGTGATAAGCATC
	PX459 *ADGRV1* intron 42 forward	CACCGCAGTATACCTCAACACTATG
	PX459 *ADGRV1* intron 42 reverse	AAACCATAGTGTTGAGGTATACTGC
**Editing efficiency amplicon**	*ADGRV1* intron 39 forward-1	TACTCCTTCCAACTGTCCCC
	ADGRV1 intron 39 reverse-1	TTCAATTACACCTCGGGCCC
	ADGRV1 intron 42 forward-1	GCCTCTCACGTGTGTATGTTG
	ADGRV1 intron 42 reverse-1	GGATTGAATGCTGACTGACCC
**Exon excision amplicon**	*ADGRV1* intron 39 forward-2	CAAACTCCTCTTTCAGTGTG
	ADGRV1 intron 42 reverse-2	TTCATACAACGCTTTACCAC
***GAPDH* loading control**	*GAPDH* exon 4 forward	CATCTTCCAGGAGTGAGTGG
	GAPDH intron 4 reverse	CCATATTGAGGGACACAAGG

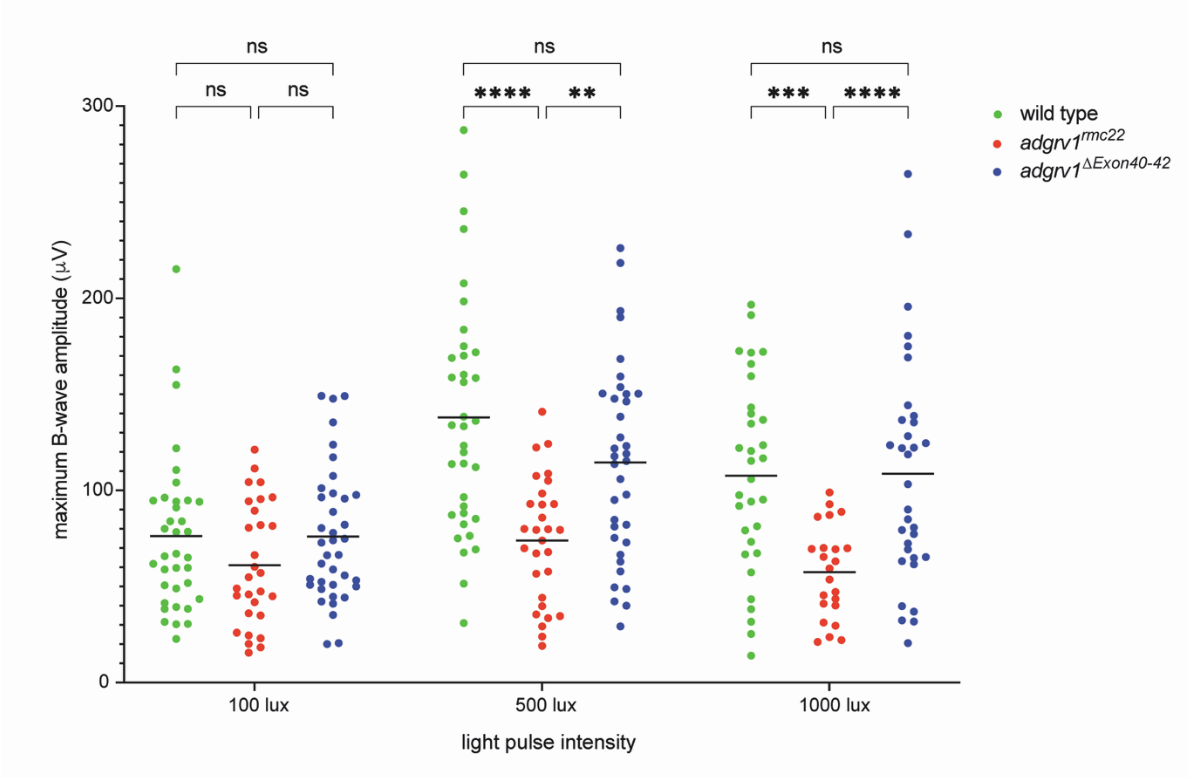

Figure S1: Electroretinogram maximum B-wave amplitudes plotted per light intensity. Maximum B-wave amplitudes following a light stimulus of 100, 500 and 1000 lux. The mean maximum B-wave amplitude of *adgrv1*$^{\Delta exon40\text{-}42}$ larvae is similar to those observed in wild types, whereas *adgrv1*^{rmc22} larvae show a decreased maximum B-wave amplitude (**, p = 0.0012; ***, p = 0.0001; ****, p < 0.0001; one-way ANOVA followed by Tukey's multiple comparison test).
